# Effect of crude polysaccharide from seaweed, *Dictyopteris divaricata* (CDDP) on gut microbiota restoration and anti-diabetic activity in streptozotocin (STZ)-induced T1DM mice

**DOI:** 10.1186/s13099-022-00512-1

**Published:** 2022-09-17

**Authors:** Nimra Zafar Siddiqui, Ata Ur Rehman, Waleed Yousuf, Asif Iqbal khan, Nabeel Ahmed Farooqui, Shizhu Zang, Yi Xin, Liang Wang

**Affiliations:** 1 Department of Biotechnology, College of Basic Medical Science, Dalian Medical University, Dalian, 116044 China; 2 Institute of Cancer Stem Cell, Dalian Medical University, Dalian, 116044 China; 3 Stem Cell Clinical Research Center, National Joint Engineering Laboratory, Regenerative Medicine Center, The First Affiliated Hospital at Dalian Medical University, No. 193, Lianhe Road, Shahekou District, Dalian, 116011 China

**Keywords:** Seaweed, *Dictyopteris divaricata*, Type-1 Diabetes Mellitus (T1DM), Polysaccharide, Streptozotocin, Inflammation, Gut Microbiota

## Abstract

Type-1 Diabetes Mellitus (T1DM) is regarded as a multifunctional, immune-related disease which causes massive destruction of islet β-cells in pancreas resulting in hyperglycemic, hypoinsulinemia and hyperlipidimic conditions. The aim of the present study, was to investigate the hypothesis that streptozotocin (STZ)-induced T1DM in Balb/c mice when treated with crude polysaccharide from seaweed, *Dictyopteris divaricata* (CDDP) depicts improvement in diabetes-related symptoms. Treatment with CDDP resulted in decreased body weight loss, improved food consumption and water intake disbalances. The CDDP effectively improved fasting blood glucose, oral glucose tolerance (OGTT), serum insulin, insulin secretion, rejuvenation of β-cells mass, serum lipid profile and pro-inflammatory cytokines levels. Additionally, treatment with CDDP increased the population of beneficial bacteria such as *Firmicutes, Bacteroidetes* and *Lactobacillus* at phylum, family and genus levels by 16S rRNA sequencing. Furthermore, immunohistological examination confirmed that CDDP reduces the inflammation and restored the structural morphology of colon and upraised the levels of insulin receptor substrate-1 (IRS*-*1), Mucin-2 (MUC-2) and tight-junction proteins (TJs) whereby maintaining the gut structures and barrier permeability. Thus, the above presented data, highlights the safe and therapeutic effects of crude polysaccharide (CDDP) from *D. divaricata* in the treatment and restoration of T1DM disorders and can be used as a food supplement alternative to diabetes medicine.

**Supplementary Information:**

The online version contains supplementary material available at 10.1186/s13099-022-00512-1.

## Introduction

Diabetes mellitus is a multifaced chronic ailment which is link with metabolic disorder of carbohydrates, proteins and fats that lead to insulin deficiency, causing hyperglycemia [1–3]. Diabetes is mainly associated with obesity [4, 5] and requires a prolong treatment to overcome its effect and complications over various parts of the body such as heart, kidney [1, 6], liver, intestine [7], eyes, neurons, teeth [8] and arteries [6]. International Diabetes Federation (IDF) in 2017, depicted diabetes as the most high-cost disease leading towards global pandemic and proclaimed that approximately 451 million individuals are diagnosed with diabetes mellitus at global scale and simultaneously, predicted that this figure shall continue to increase drastically to 693 million by 2045 [8–11].

The total diabetic cases documented around the globe are approximately 5–10% which encompasses by type-1 diabetes mellitus (T1DM) [9]. Although vast correspondence has represented the upsurge in T1DM dissemination in developed regions after World War II. Moreover, T1DM cause protracted disease onset which is associated with immune system and thus, the destruction was ensued by 80% of β-cells in the islets of pancreas [12], which eventually effects the insulin uptake to acquire the following conditions i.e., hyperglycemia [13], hyperlipidemia, inflammation, oxidation [6] and insulitis [4]. While several other factors which augmenting in T1DM cases escalation usually affect young entities include; the use of antibiotics, immunopathogenesis [14], familial history [15], genetic and environmental factors mainly, diet and unhealthy lifestyle [1, 12, 16, 17].

For many decades *in-vivo* subjects were injected with various chemical compounds to observe the surge of diabetes mellitus and its physiological effects [18, 19]. Currently, streptozotocin (STZ) is the utmost drug to induce diabetes [20–22]. The utilization of reduce concentration of STZ (i.e., low-doses) in manifold pattern is now widespread and studied robustly in animal models because of its stability, expediency and also it bears a resemblance to T1DM as in humans. The progressive events of T1DM appeared by application of STZ in mice was endeavour to severe complications which include inflammation of pancreatic islet, insulin deficiency by toxicity in β-cells by GLUT-2 (glucose transporter-2), presence of autoantibodies and insulitis [20, 22, 23].

As stated in reported literature, various insulin injectables and distinct anti-diabetic compounds in the form of oral contraceptive and injections are available for the treatment of diabetes but does not provide complete cure because of side effects and cost compromise remedy [2, 24]. Although metformin, a biguanides, is effectively used for the treatment of type-1 and type-2 diabetes by inhibiting the indigestion of carbohydrates and activity of α-glucosidase, in turn lowers the blood glucose, increase the sensitivity of insulin whereby reducing the production of glucose by liver. However, persistent use diabetic drugs may lead to the development of lactic acidosis, diarrhoea and liver problem [25–27]. Therefore, in order to conquer the knock-on effect and economic burden of diabetes, a variety of natural products are proposed such as probiotics and prebiotics, used to treat diabetes mellitus in all age of individuals [28]. Certain possibilities are available for the isolation of these natural substances in the form of polysaccharides from microorganisms, plants, animals and mushrooms [7, 29, 30]. More interestingly this necessary form of prebiotics had showed significant results in order to degenerate the pathogenesis of T1DM with objective to subjugate different mechanisms like dysfunction of β-cells, enhancement of insulin and inhibition of glucosidase and α-amylase [9, 28, 31]. The prebiotics utilization had also proven to transform and restored gut microbiome in positive aspect [32].

The contemporary extended sufficient data regarding continuously expanding number of ailments has driven the attention of scientific domain towards exploration of gut microbiota and its barriers. In the current epoch, the necessity of gut microbiota has become an essential part in the regulation and developmental mechanism of distinctive disorders to support promising outcome of the therapy [10, 32, 33]. These gut microbiotas not only aids in metabolites production but also effect the food fermentation as a result the gut permeability and immune status of a host compromised which brings changes in the bacterial population and function [34]. All these changes may contribute in the bacterial dysbiosis, eventually leading to the development of T1DM [35, 36]. In harmony, the concrete evidence had showed a close interface between diabetes and gut microflora [31, 37, 38]. Although these gut microflora benefits the host in many ways such as controlling body weight, hormones, activity of proinflammatory cytokines and hyperglycemia [33].

Polysaccharides from many organic products are getting considerable attention in recent years because of their low cost, less toxicity and effective potential in treating diabetes are promising factors [24]. The naturally isolated polysaccharides from marine organisms had demonstrate substantial effect against diabetes [10, 28]. They usually cover about 80% of the biological resources of the earth as they can tolerate extreme conditions of sea including pH, salinity, water currents and pressures [39, 40]. Being an excellent source of proteins [41], vitamins and minerals they are widely studied over the past decades [40, 42]. A micro alga include seaweed, *Dictyopteris divaricata,* a brown alga distributed along the coastline of Yellow Sea, China, belongs to a family *Dictyotaceae,* known as Okamura (basionym: *Haliseris divaricata Okamura*) [43, 44], has been used as a source of functional food and therapeutic agent in China [40]. *Dictyopteris divaricata* was broadly investigated for its chemical composition as it has various number of functional compounds [39, 43, 45, 46]. *D. divaricata* ethanol and ethyl acetate extract was examined for its anti-cancer activity in eukaryotic cell lines [47] and dichloromethane/methanol and ethanol extracts for α-Glucosidase inhibition activity which can be choice as remedy for diabetes, obesity and hyperlipoproteinemia [48, 49]. Furthermore, auxiliary elements of extracts also retain quite remarkable biological activities such as anti-inflammatory, anti-oxidant [39], anti-tumor, anti-microbial, anti-allergic activity [40, 43, 50] and neuroprotective effects [51].

However, to the best of our knowledge, there is no such studies have been done on activity of its crude polysaccharide against STZ-induced T1DM and its effect on gut barrier and gut microbiome. Therefore, the current study was used to see the curative effects on the reduction of polydipsia and polyphagia conditions, hyperglycemia, hyperlipidemia, pro-inflammatory cytokines levels, oxidative stress markers and alleviation of body weights and serum insulin levels. In addition, restoration of gut barrier permeability and gut microbiota dysbiosis occurred by streptozotocin to induce T1DM was also studied.

## Materials and methods

### Chemical and reagents

The seaweed *Dictyopteris divaricata,* was purchased in the form of dry powder from Dalian, Liaoning, China. The stool DNA isolation kit (FORGENE) was obtained from Chengdu, China. The bicinchoninic acid (BCA) protein kit and serum insulin, interleukin-1β (IL-1β), interleukin-2 (IL-2), interleukin-6 (IL-6), tumour necrosis factor-alpha (TNF-α), interferon-gamma (IFN-γ) and Malondialdehyde (MDA): Mouse ELISA kits were purchased from TransGen Biotech Co, Ltd., Beijing, China, and Shanghai Longton Biotechnology Co, Ltd., Shanghai, respectively. Serum Super Oxide Dismutase (SOD) kit was purchased from Solarbio Life Sciences (catalog no. BC0170). The primary antibodies [goat antirabbit: β-actin, Mucin-2 (MUC-2), Claudin-1, Occludin, Zonula occludens-1 (ZO-1) and insulin receptor substrate-1 (IRS-1)], secondary antibodies and the radioimmunoprecipitation assay (RIPA) buffer were purchased from Proteintech (Wuhan, China). The DAB substrate chromogen system and horseradish peroxidase-conjugated secondary antibody were from ZSGB-BIO (Beijing, China). Streptozotocin (STZ) was purchased from Sigma Chemical Co. (St. Louis, MO, USA) and stored at -20 ℃ freezer. All the other chemicals used in the experiments were of highest analytical grade and purchased commercially.

### Crude polysaccharide (CDDP) extraction from the seaweed, *Dictyopteris divaricata*

The extraction of crude polysaccharide (CDDP) from seaweed *Dictyopteris divaricata,* was executed according to previously reported study [89]. Briefly, the dried powder form of seaweed was passed through a mesh sieve (0.42 mm) and stored in an air-tight container. The powder was mixed with distilled water in a w/v ratio (1 g/50 ml), boiled at 70 ℃ for 3 h. The mixture was slightly cooled, proteins were removed by trichloroacetic acid (TCA) in a concentration of 1.5% (v/v) and then pH was adjusted to neutral (pH:7) with 2 M sodium hydroxide (NaOH). The mixture was centrifuged at 5000 rpm for 10 min to form pellets which contains protein in a coagulated form. The supernatant was collected and evaporated at 60 ℃ in a continuous rotation using a rotary evaporator. The protein concentration was measured by BCA (bicinchoninic acid) assay kit as per manufacturer instructions. Three times the final volume of the mixture, 99.7% ethanol was added, mixed and kept at 4 ℃ for 12 h. Mixture was centrifuged at 5000 rpm for 10 min. The pellet was collected as crude polysaccharide of *Dictyopteris divaricata* (CDDP) and dried using a freeze-drying vacuum technique.

### Characterization of crude polysaccharide (CDDP) and sugar content

The composition of monosaccharide and molecular weight of crude polysaccharide (CDDP) was determined by using HPLC (high-performance liquid chromatography) technique [90]. The carbohydrate content was determined by the phenol-H_2_SO_4_ method [91].

### Animal housing

Healthy male Balb/c mice (18 ± 2 g, 5–6 weeks old) were acquired from Specific-pathogen-free (SPF) animal care centre with the approval from Dalian Medical University, Dalian, China. Mice were permitted to adapt themselves in a hygienic and ventilated laboratory environment for about 1 week prior to the start of the experimental procedures at a temperature 20 ± 3 ℃ and 55 ± 10% relative humidity with a cycle of 12/12 h day/night rhythm. Preliminary, measurement of body weight and blood glucose level were determined before initialization of the experiment. All the mice were fed with standard laboratory chow diet (Jiangsu Medison Biomedical Co., Ltd., Yangzhou, Jiangsu Province, China) with free access to water throughout the duration of the study. The study was carried out according to the strict protocols of use and care of laboratory animals by our Institutional Committee. All the experimental procedure was approved by Animal Care and Research Ethics Committee Dalian, China.

### Induction of type-1 diabetes in experimental mice

**Fig. 1:**
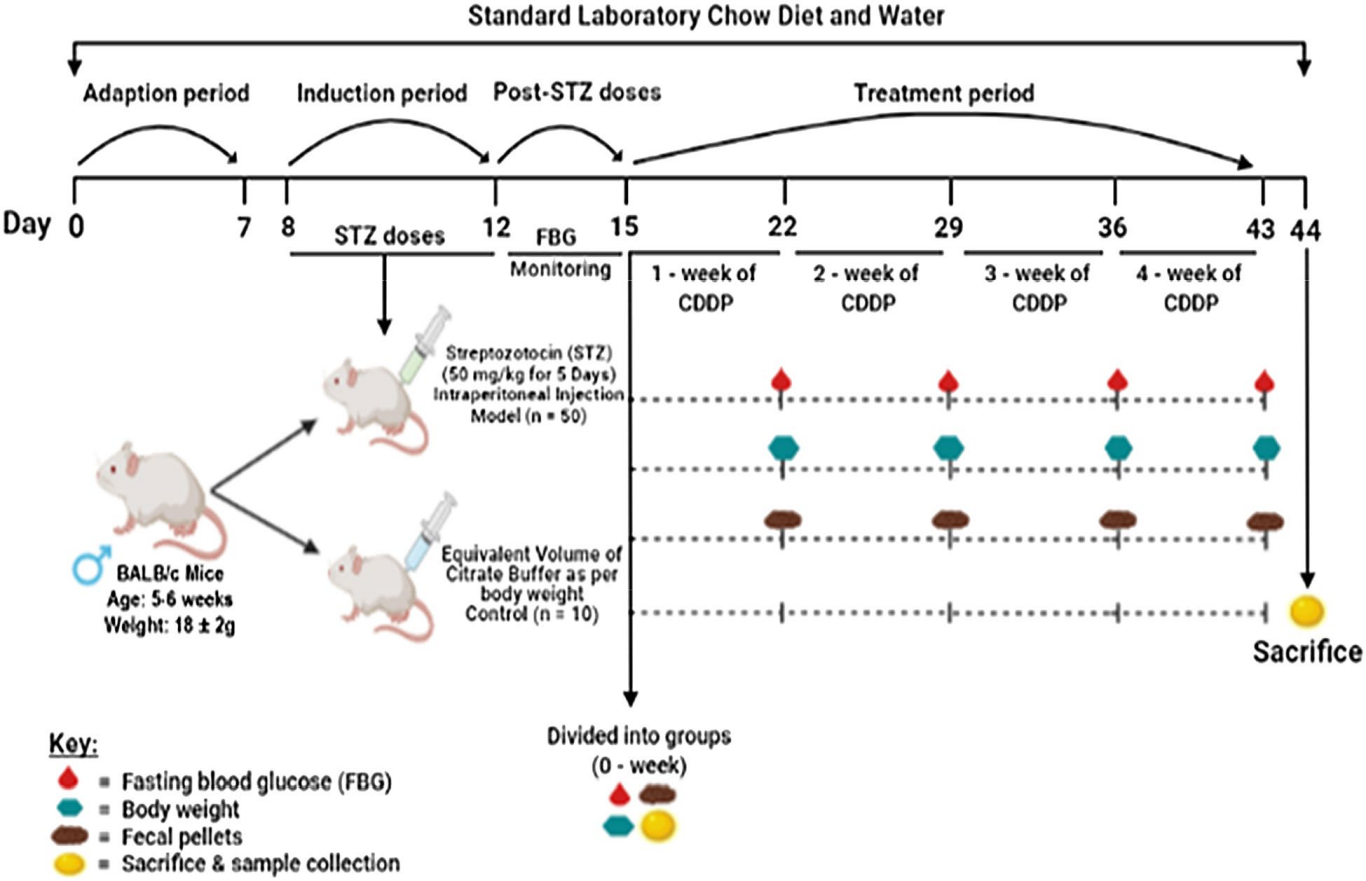
Schematic representation of study design. STZ-induced T1DM (model) mice were given normal laboratory chow diet and normal water throughout the experiment. Adaptation to laboratory environment (day 0 to day 7), followed by intraperitoneal injection of STZ (50 mg/kg body weight) to overnight fasted mice for 5-consecutive days (day 8 to day 12). The mice were divided into six groups (n = 10 mice in each group): control group (normal healthy control), model group (STZ-induced T1DM), metformin group (200 mg/kg body weight), DDP-Low: low dose of CDDP (200 mg/kg body weight), DDP-Med: medium dose of CDDP (400 mg/kg body weight) and DDP-High: high dose of CDDP (600 mg/kg body weight) after the monitoring for 3-days (day 13 to day 15) for diabetes development. From day 16 to day 43 (week 1 till week 4), the mice were orally gavage with crude polysaccharide (CDDP) and metformin HCl. Weekly collection of feces, followed by record of fasting blood glucose levels, body weights, food consumption and water intake for 4-weeks of the study duration

A total of sixty mice [60] were included in the study, out of which n = 10 was considered as healthy normal control (control group) and n = 50 as STZ-induced T1DM group (model group). After being quarantined for one week the mice were fasted for about 6 h prior to the induction, the T1DM model mice was given an intraperitoneal injection of streptozotocin, freshly prepared in 0.1 M of ice-cold citrate buffer (pH 4.5) at a dose of 50 mg/kg body weight within 15 min of preparation for 5 consecutive days (once every day from day 8 to day 12), while control group mice were injected with similar volume of citrate buffer as that of model group [92–94]. Subsequently, following day after STZ injection the mice were given drinking water with 10% sucrose till 5 days of STZ doses, to avoid the mortality that might occur because of excess loss of insulin from pancreas. On day 13 (day 1 of post-STZ dose), onwards the mice were given normal drinking water without inclusion of 10% sucrose [23]. The blood glucose levels of mice were detected after 72 h (i.e., day 15) of last STZ injection using a standard glucometer (Sinocare Inc., Hunan, China) through a blood drop from a tail vein placed on a blood glucose test strip (Sinocare Inc., Sanyo Biosensors Inc.). The body weight of control and model (T1DM) group, both pre and post STZ injection along with body weight gain/loss after STZ injection had been enlisted in Additional file 1: Table: S1. Mice with a blood glucose level higher than 11.1 mmol/L or > 200 mg/dL were considered as diabetic mice. The treatments were started on day 16 and continued till day 43. The illustration of the design for the drug delivery and establishment of T1DM was summarized and presented in Fig. 1.

**Fig. 2:**
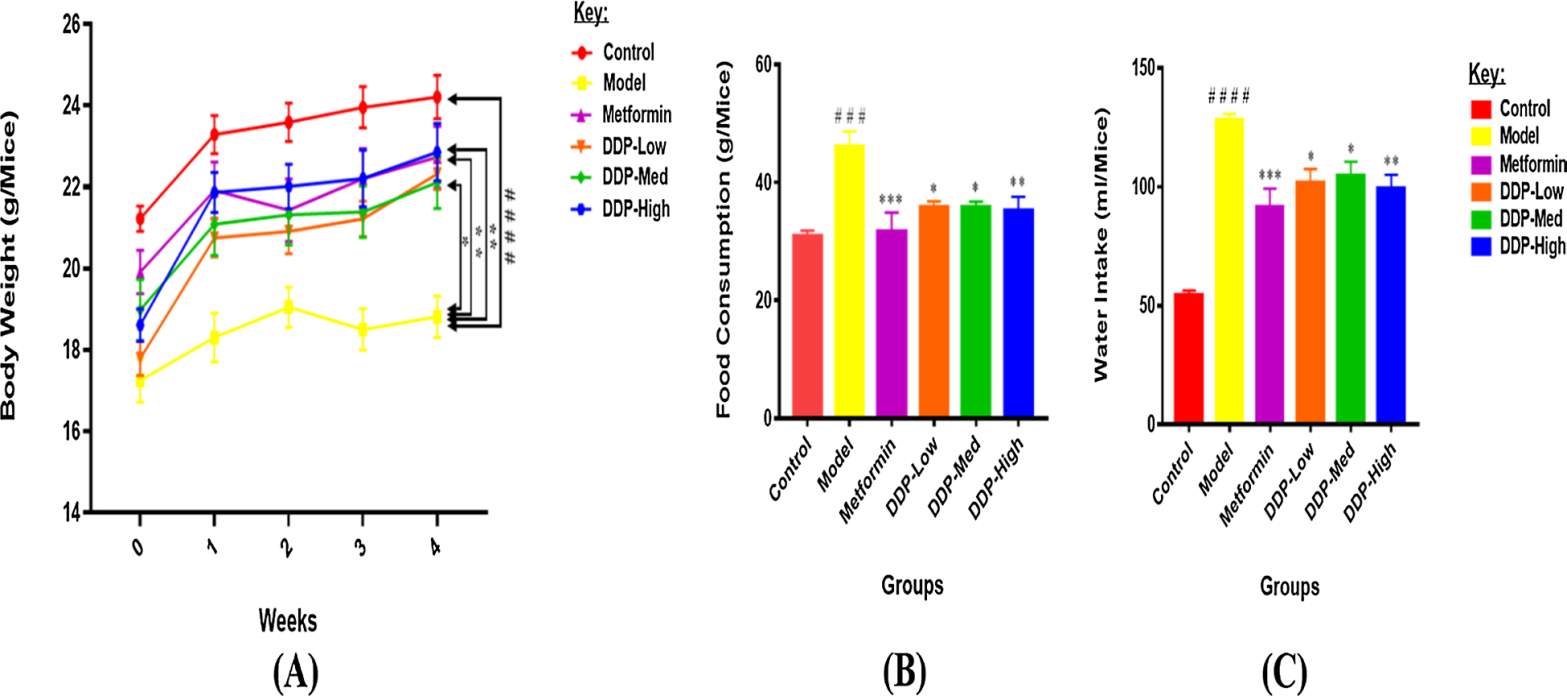
Effect of crude polysaccharide (CDDP) on: **A** Body weights (g), **B** Food Consumption (g) and **C** Water Intake (ml) of STZ-induced T1DM mice measured once in a week for 4-weeks during oral administration of CDDP treatment. Model group showed reduced body weights and increased food/water intake when compared to control group. Mice receiving CDDP doses and metformin via oral route had significantly elevated the body weights and reduced the consumption of food and intake of water in comparison with model group. Data were represented as Means ± SEM (n = 10), evaluated by one-way ANOVA by Tukey’s Multiple Comparison Test. Control group (normal healthy control), model group (STZ-induced T1DM), metformin group (200 mg/kg body weight), DDP-Low: low dose of CDDP (200 mg/kg body weight), DDP-Med: medium dose of CDDP (400 mg/kg body weight) and DDP-High: high dose of CDDP (600 mg/kg body weight). # p < 0.05, ## p < 0.01 ### p < 0.001 and #### p < 0.0001, model group compared with control group * p < 0.05, ** p < 0.01 and *** p < 0.001, CDDP and metformin treatment groups compared with model group

**Fig. 3:**
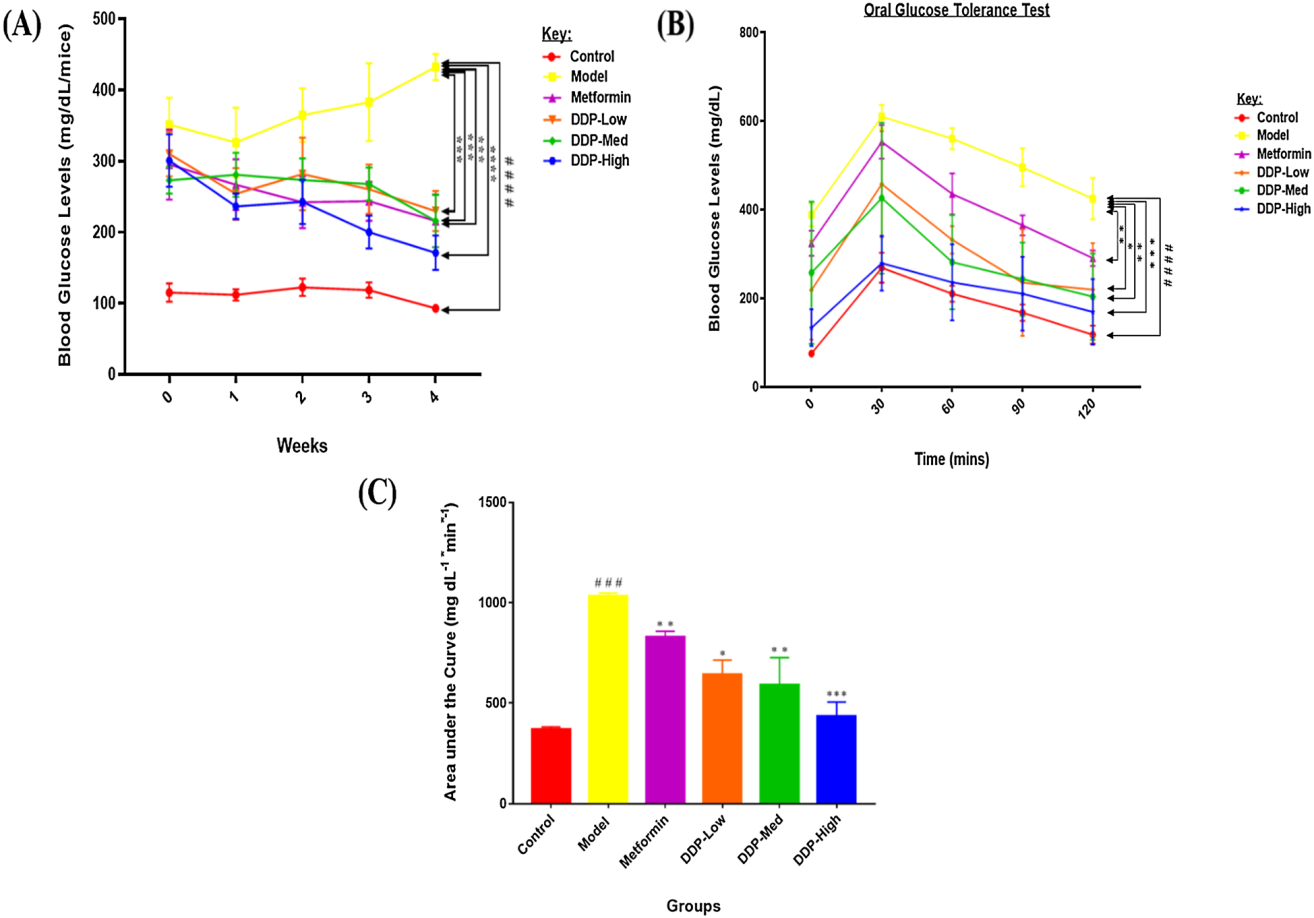
Crude polysaccharide (CDDP) depreciate hyperglycemia and 2-h postprandial blood glucose (PBG-2 h) in STZ-induced T1DM mice. **A** Fasting blood glucose levels (mg/dL) in mice were measured at 0, 1, 2, 3 and 4 weeks during oral dosing of CDDP treatment. Model group showed an elevated levels of fasting blood glucose when compared to control group. Mice receiving CDDP doses and metformin via oral route had significantly reduced the elevated fasting blood glucose levels in comparison with model group. **B** Oral administration of 2.0 g/kg body weight of glucose to all overnight fasted mice. Measurement of blood glucose (mg/dL) was done at 0, 30, 60, 90 and 120 min. **C** AUC (mg/dL) was calculated by the formula. Data were represented as Means ± SEM (n = 10), evaluated by one-way ANOVA by Tukey’s Multiple Comparison Test. Control group (normal healthy control), model group (STZ-induced T1DM), metformin group (200 mg/kg body weight), DDP-Low: low dose of CDDP (200 mg/kg body weight), DDP-Med: medium dose of CDDP (400 mg/kg body weight) and DDP-High: high dose of CDDP (600 mg/kg body weight). # p < 0.05, ## p < 0.01 ### p < 0.001 and #### p < 0.0001, model group compared with control group * p < 0.05, ** p < 0.01, *** p < 0.001 and **** p < 0.0001, CDDP and metformin treatment groups compared with model group

**Fig. 4:**
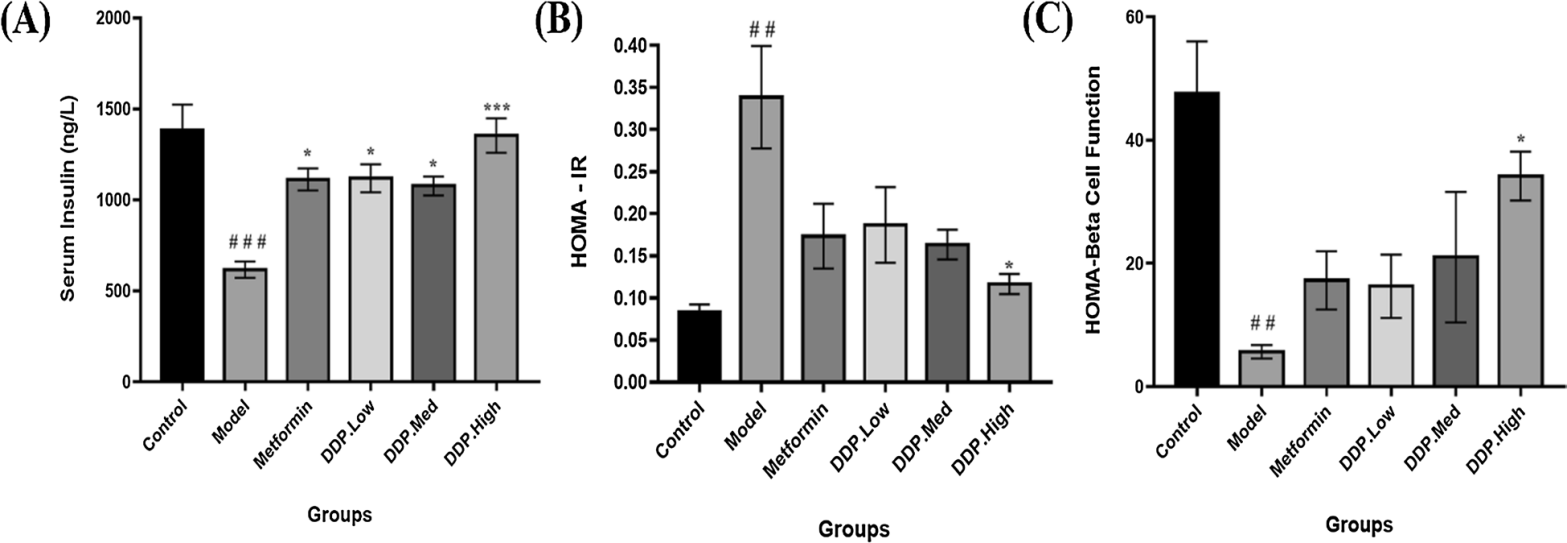
Changes in the serum insulin levels, HOMA-IR and HOMA-β-cells function of the mice followed by 4-weeks of CDDP treatment. **A** Serum insulin levels. Model group showed the lower insulin levels which was enhance after CDDP and metformin treatments. **B** HOMA-IR. **C** HOMA-β cell function. Data were represented as Means ± SEM (n = 6), evaluated by one-way ANOVA by Tukey’s Multiple Comparison Test. # p < 0.05, ## p < 0.01 and ### p < 0.001, model group compared with control group * p < 0.05, ** p < 0.01 and *** p < 0.001, CDDP and metformin treatment groups compared with model group

**Fig. 5:**
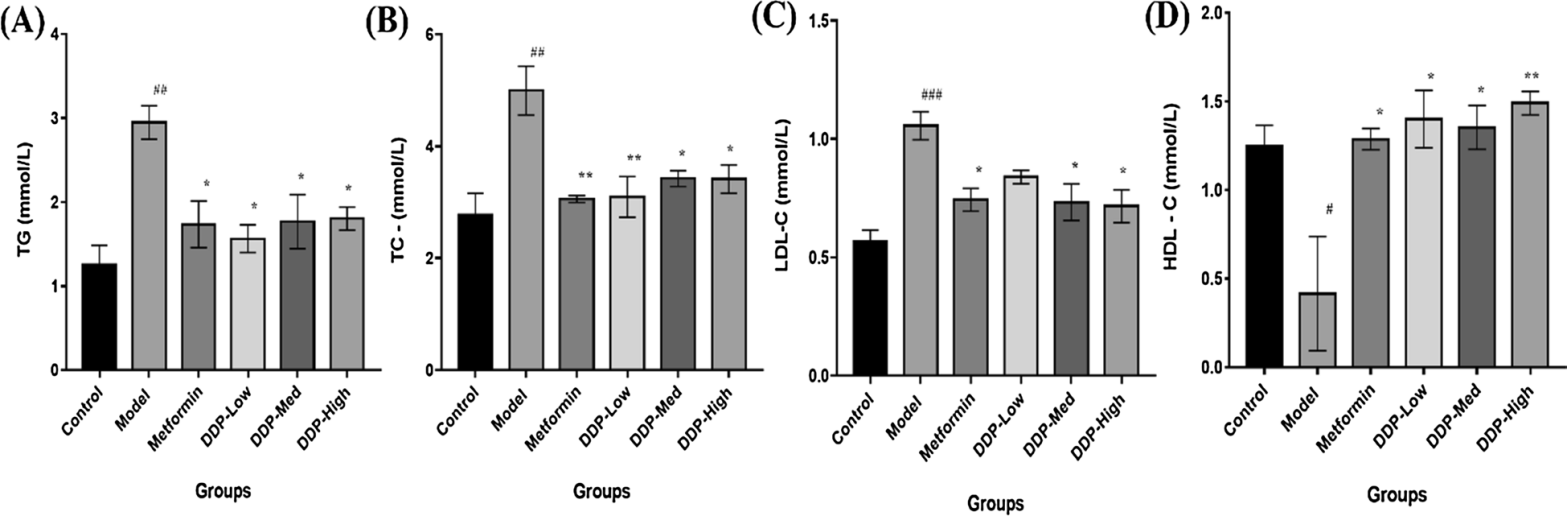
Amelioration of dyslipidemia by crude polysaccharide (CDDP) in STZ-induced T1DM mice. Levels of: **A** TG, **B** TC, **C** LDL-C, and **D** HDL-C were determined at the end of the experiment. Data were represented as Means ± SEM (n = 6), evaluated by one-way ANOVA by Tukey’s Multiple Comparison Test. # p < 0.05, ## p < 0.01 and ### p < 0.001, model group compared with control group * p < 0.05 and ** p < 0.01, CDDP and metformin treatment groups compared with model group

**Fig. 6:**
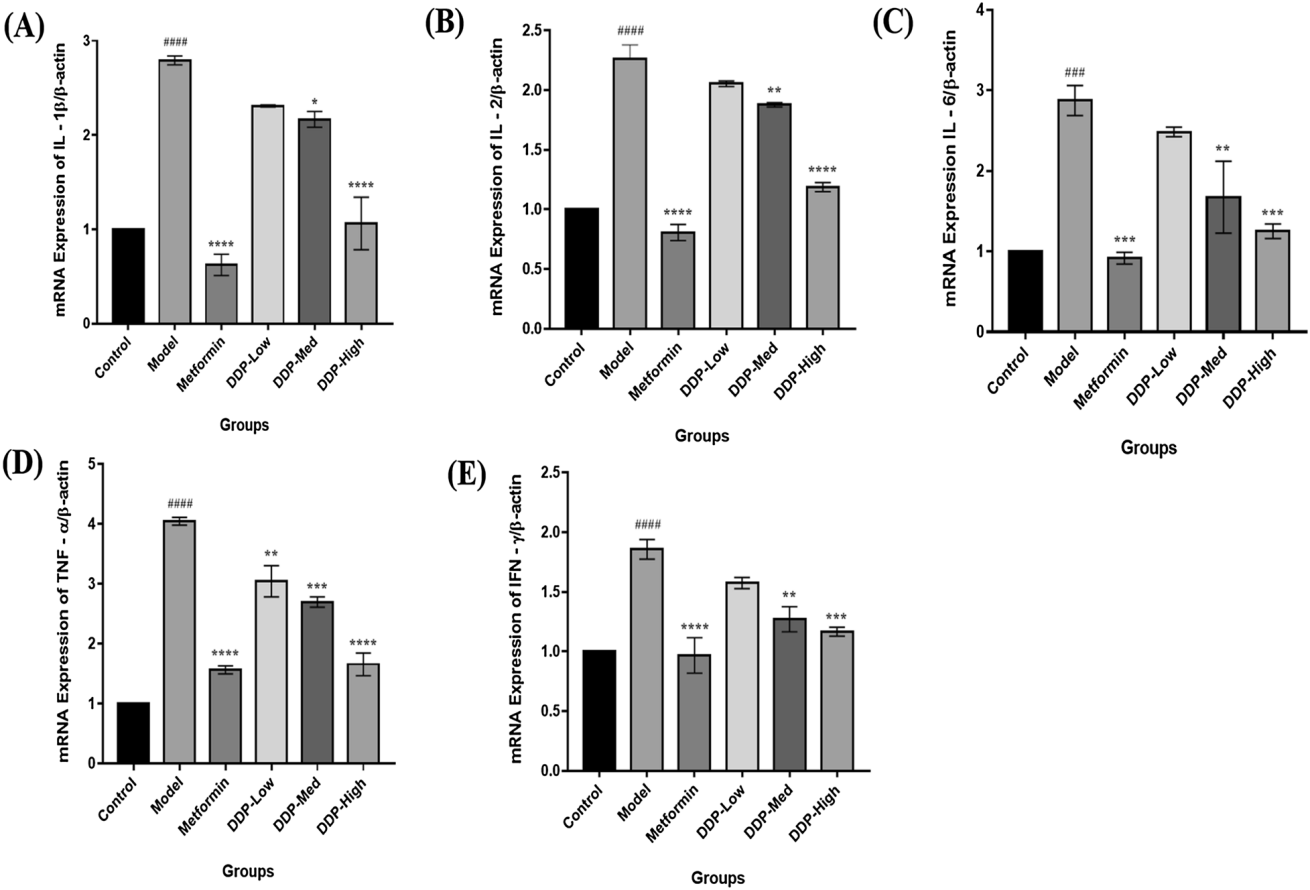
Effect of crude polysaccharide (CDDP) on pro-inflammatory cytokines levels in STZ-induced T1DM mice. The analysis was carried out after the oral administration of CDDP and Metformin for 4-weeks. Levels of: **A** IL-1β, **B** IL-2, **C** IL-6, **D** TNF-α, and **E** IFN-γ by qPCR were determined at the end of the experiment. Data were represented as Means ± SEM (n = 6), evaluated by one-way ANOVA by Tukey’s Multiple Comparison Test. # p < 0.05, ## p < 0.01, ### p < 0.001 and #### p < 0.0001, model group compared with control group * p < 0.05, ** p < 0.01, *** p < 0.001 and **** p < 0.001, CDDP and metformin treatment groups compared with model group

**Fig. 7:**
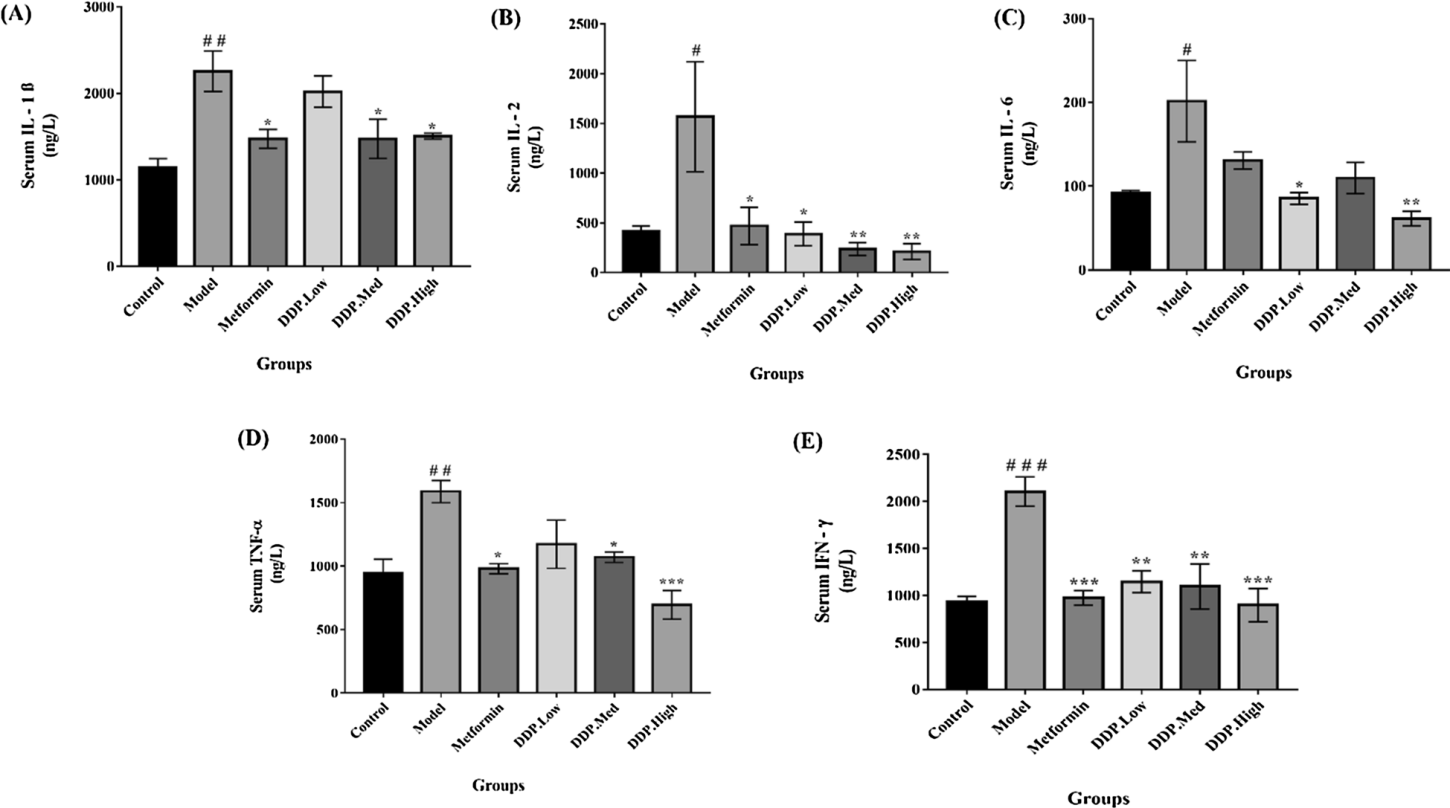
Quantification of pro-inflammatory cytokines levels in STZ-induced T1DM mice. The analysis was carried out after the oral administration of CDDP and Metformin for 4-weeks. Levels of: **A** IL-1β, **B** IL-2, **C** IL-6, **D** TNF-α, and **E** IFN-γ by ELISA were determined at the end of the experiment. Data were represented as Means ± SEM (n = 6), evaluated by one-way ANOVA by Tukey’s Multiple Comparison Test. # p < 0.05, ## p < 0.01 and ### p < 0.001, model group compared with control group * p < 0.05, ** p < 0.01 and *** p < 0.001, CDDP and metformin treatment groups compared with model group

**Fig. 8:**
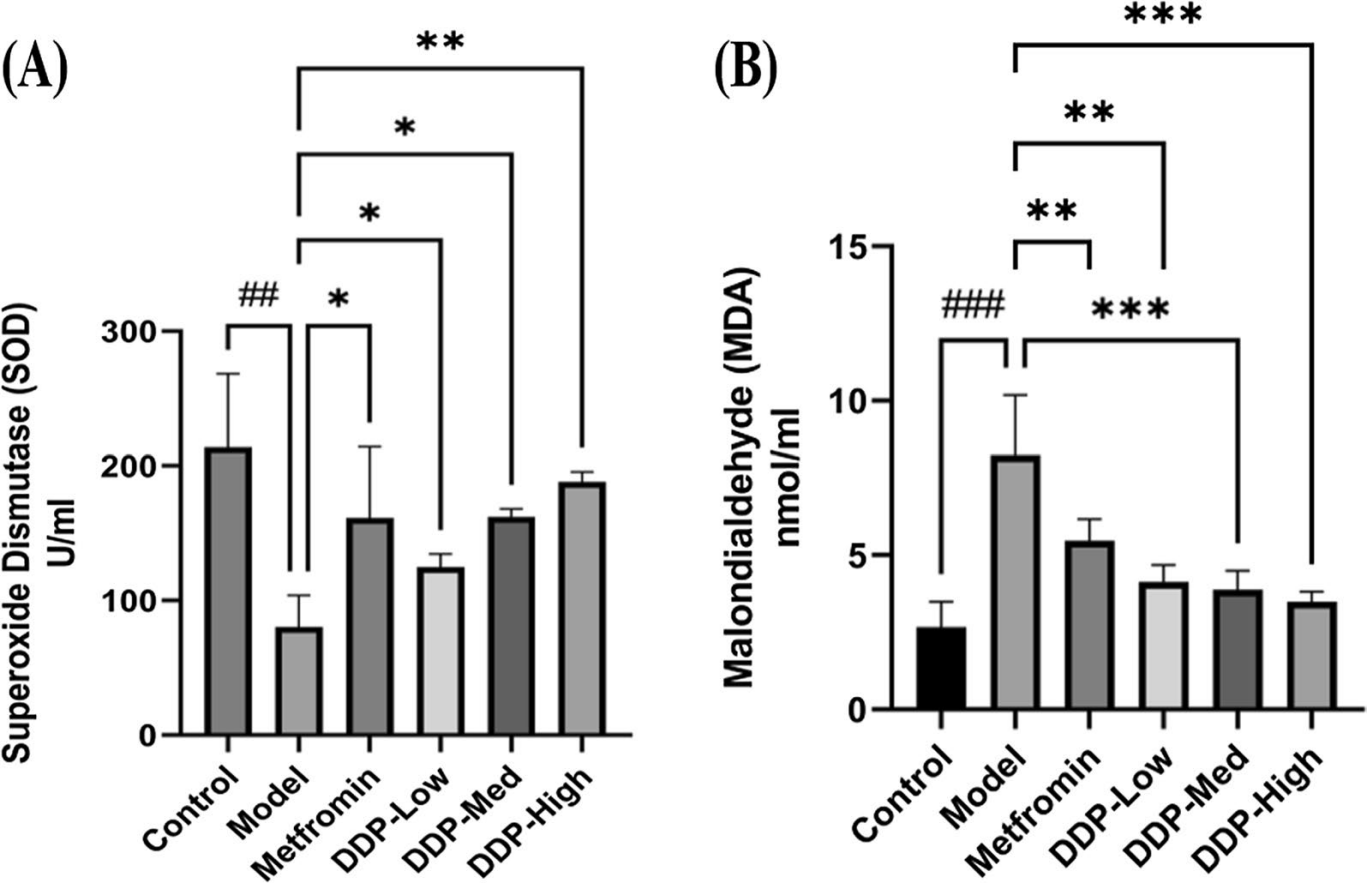
Effect of CDDP on oxidative stress markers after 4-weeks of oral treatment. The levels of: **A** SOD and **B** MDA in serum were analyzed. Data were represented as Means ± SEM (n = 6), evaluated by one-way ANOVA by Tukey’s Multiple Comparison Test. # p < 0.05, ## p < 0.01 and ### p < 0.001, model group compared with control group * p < 0.05, ** p < 0.01 and *** p < 0.001, CDDP and metformin treatment groups compared with model group

**Fig. 9:**
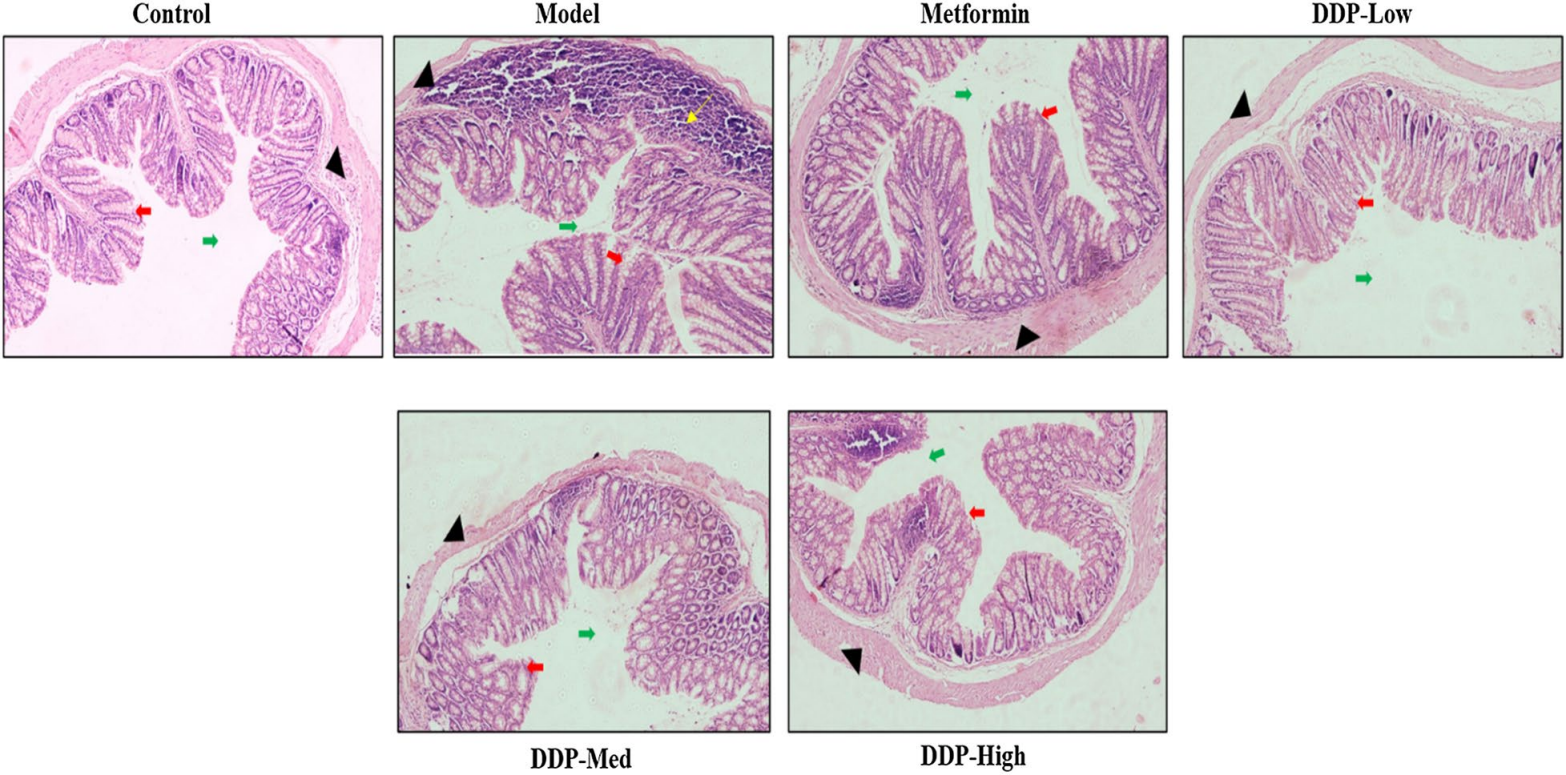
Histochemical analysis [H & E] of: colon of STZ-induced T1DM mice and effect of CDDP and metformin treatment groups. Epithelial cells (represented by black arrowhead), epithelial cells and goblet cells (represented by red arrow), inflammatory cells (represented by yellow arrow) and mucosal surface (represented by green arrow). The picture is a representative of the pancreas and colon from control, model, metformin and CDDP treatment groups (DDP-Low, DDP-Med and DDP-High) mice

**Fig. 10:**
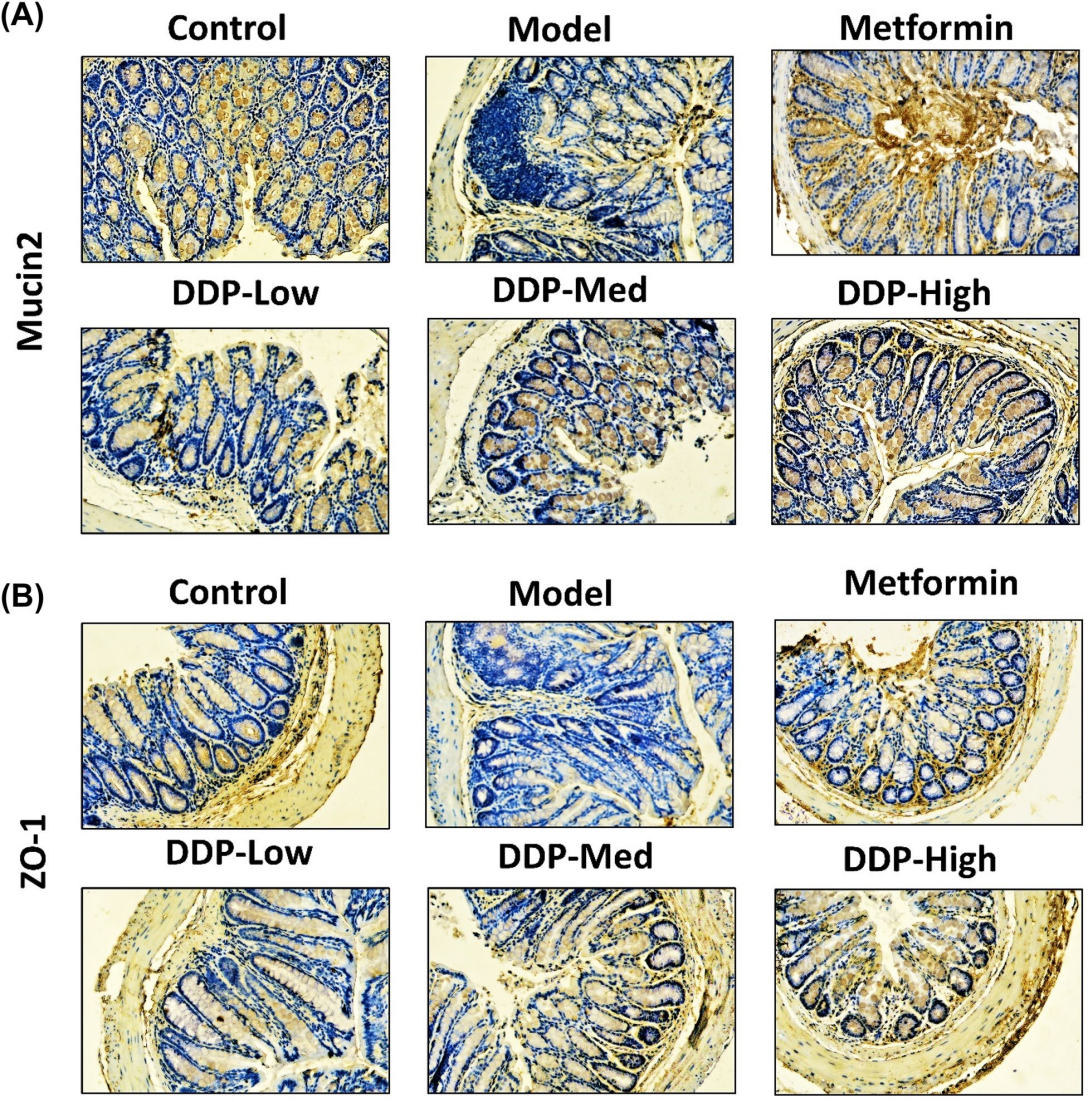
Crude polysaccharide (CDDP) enhanced the expression of: **A** Mucin-2 (MUC-2) and **B** Zonula occludens-1 (ZO-1) in STZ-induced T1DM mice colon by immunohistochemistry technique after 4-weeks treatment. Inflammatory cells (demonstrated by red arrow), goblet cells (demonstrated by yellow arrow). Mucin expression (demonstrated by green arrow) and Zonula occludens-1 expression (demonstrated by orange arrow) were shown. The picture is the representative of colon of six different treatment groups. Original magnification: 20x, scale bar: 100 µm

**Fig. 11:**
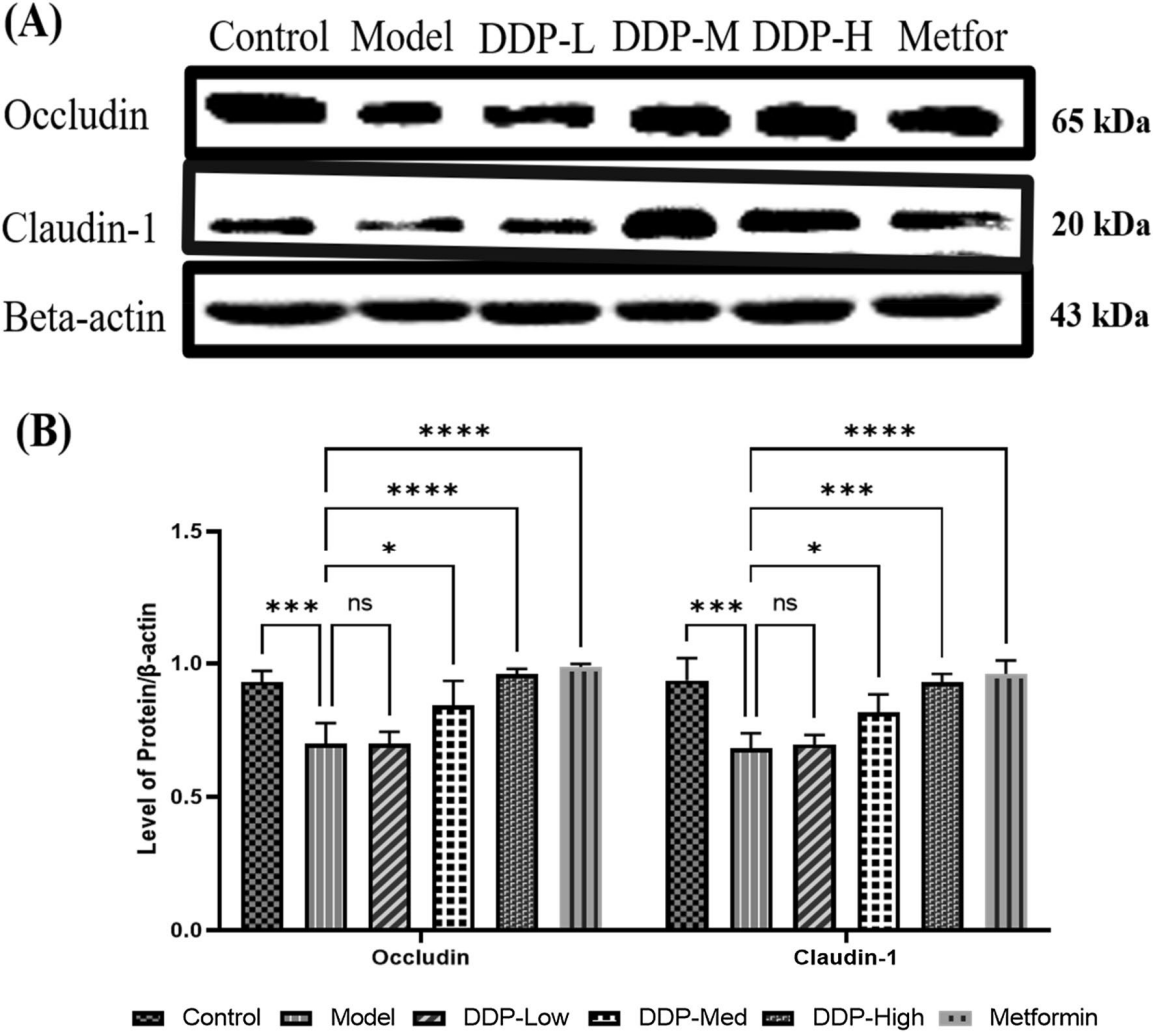
Effect of CDDP treatment on tight junction proteins (TJs) expression in the colon epithelium. **A** The relative expression of Occludin and Claudin-1 in STZ-induced T1DM mice were analyzed by western blotting technique after 4-weeks treatment using β-actin as an internal control. Western blots are the representative of three experiments. **B** Bar graph represent the intensity of relative protein band against β-actin as an internal control, quantified by NIH image J software. Data obtained from three experiments are presented as Mean ± SEM (n = 6). * p < 0.05, ** p < 0.01, *** p < 0.001 and **** p < 0.001, model group compared with control group and CDDP and metformin treatment groups compared with model group

**Fig. 12:**
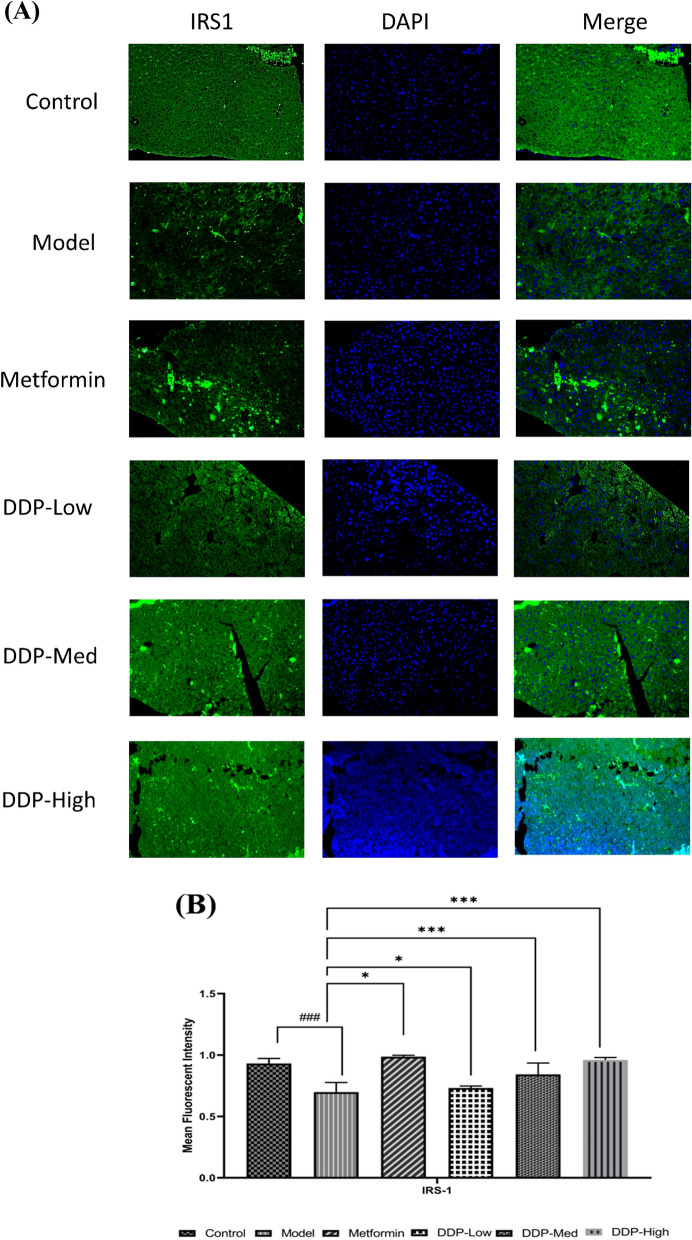
**A** Immunofluorescent staining of insulin receptor substrate-1 (IRS-1) in the pancreas of STZ-induced T1DM mice. Original magnification: 20x, scale bar: 100 µm. **B** Quantification graph of Immunofluorescent staining (IRS-1)

**Fig. 13:**
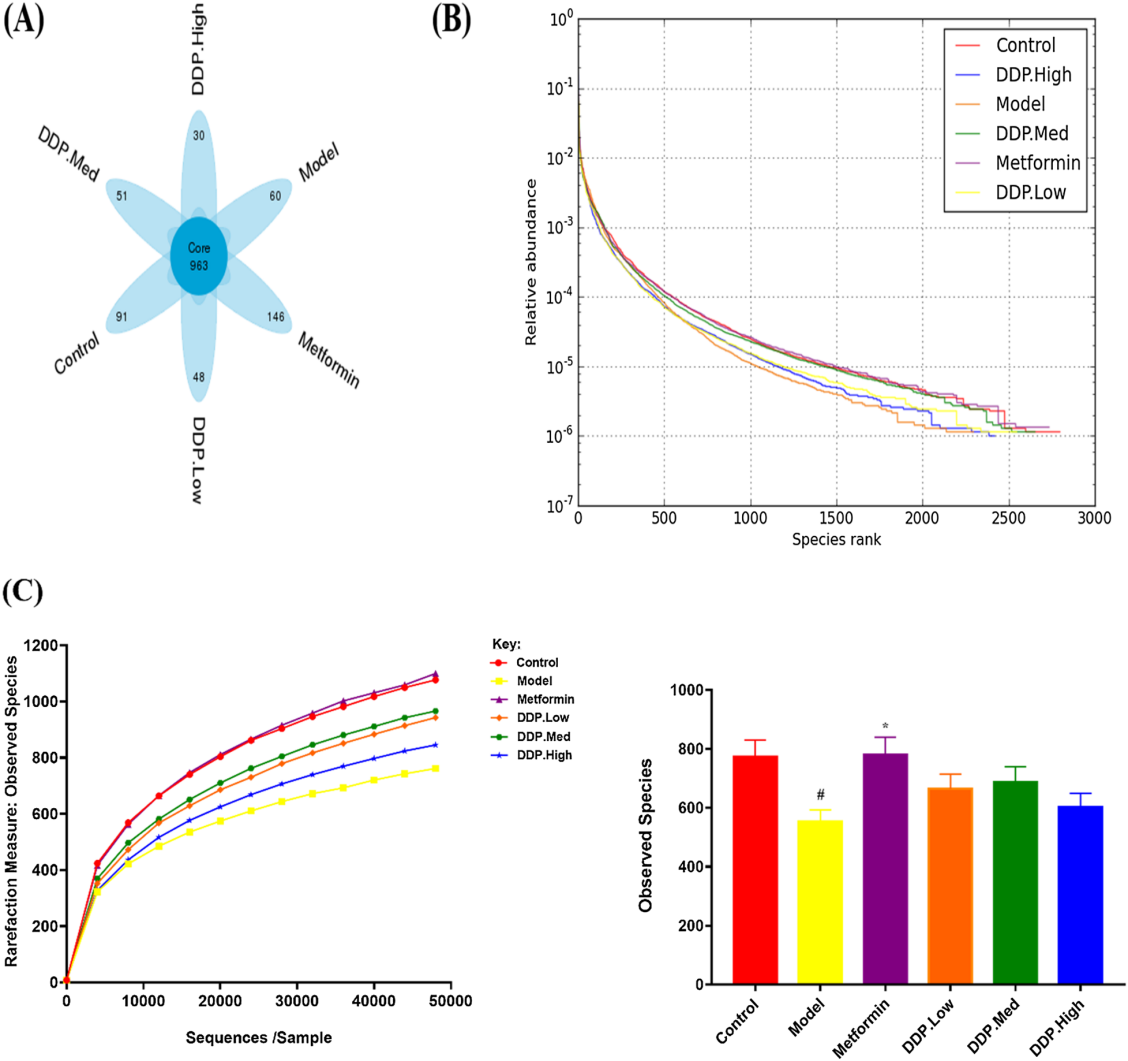
**A** Venn Diagram. Demonstrating the OTUs among six groups. **B** Rank abundance curve. Highlights the evenness, abundance and diversity of species. The x-axis represents the number of OTUs (Operational Taxonomic Units) in the sample and y-axis represents the number of species in the sample. **C **Rarefaction curve: Observed Species, represents the species diversity and abundance. gfd

**Fig. 14:**
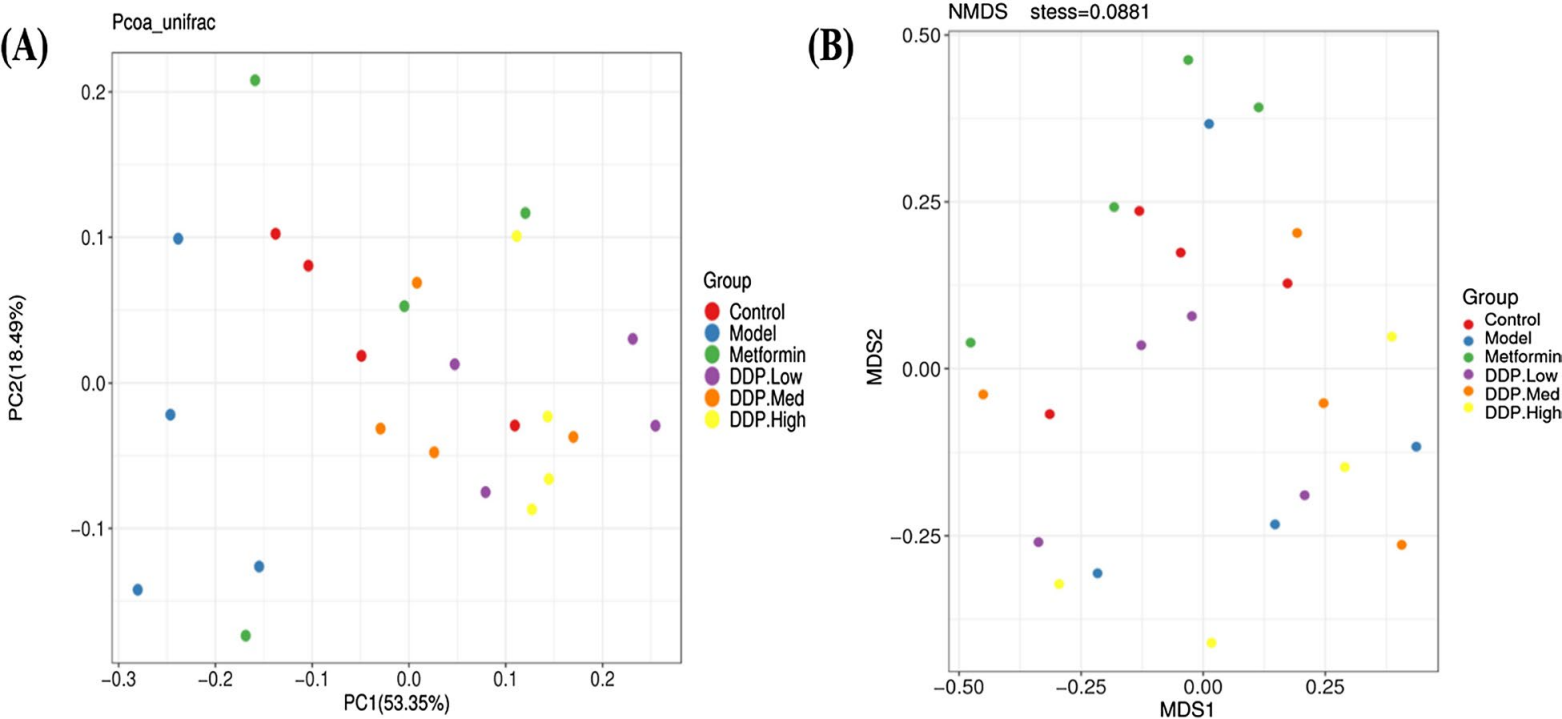
The output of the graphical representation of beta (β)-diversity. **A** PCoA (Principal coordinate analysis) plot. **B** NMDS (Non-metric multidimensional scaling) plot with weighted-unifrac distances, represents each sample by a dot in the graph presented by different colors assigned for each treatment groups. Closer points depict the similarity; distant the points more the dissimilarity

**Fig. 15:**
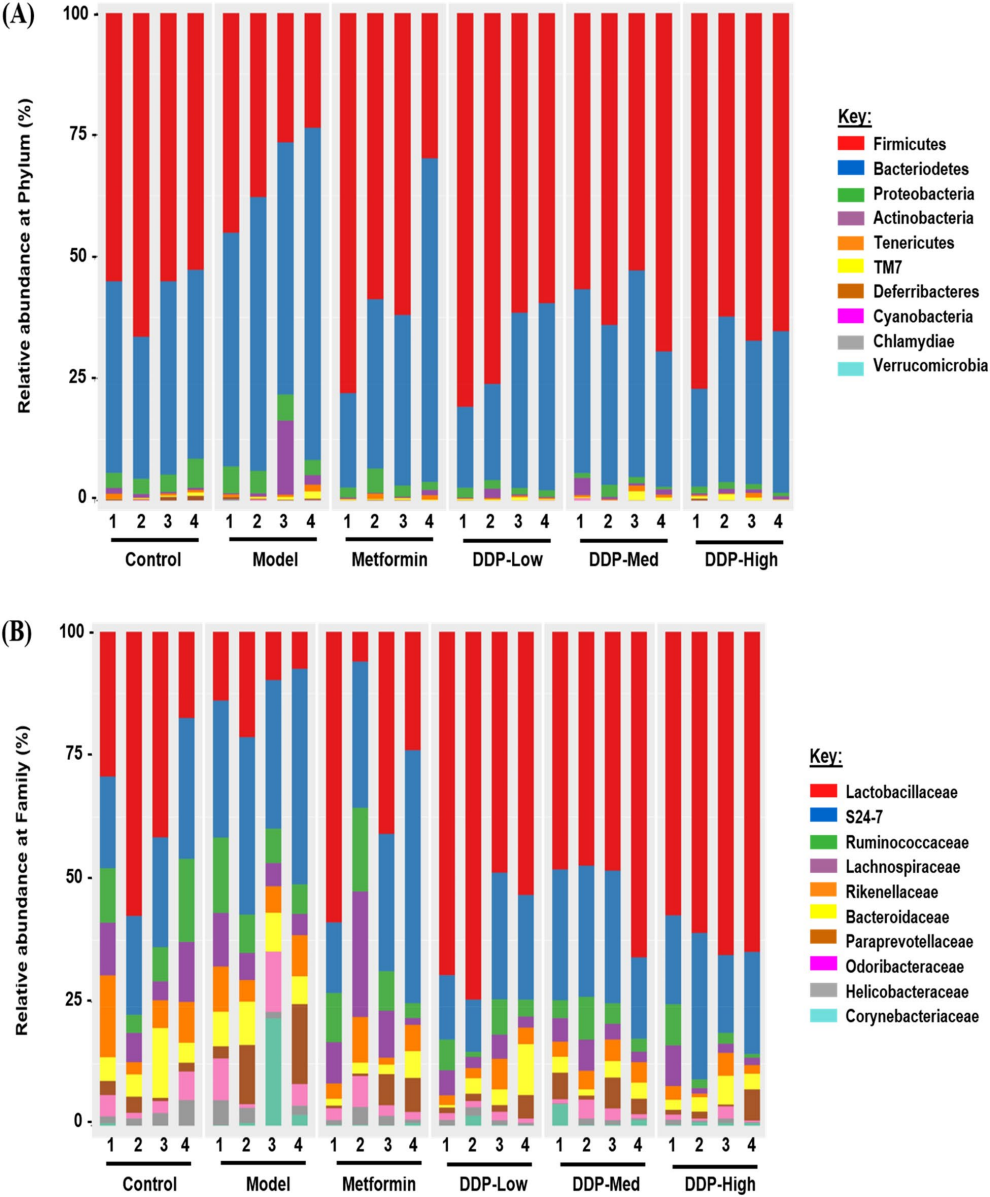
Relative abundance of microbial population (%) at: **A** Phylum level (n = 4). **B** Family level (n = 4)

### Experimental design and procedure

Concisely, after successful development of diabetes, the STZ-induced T1DM mice were divided into six groups (n = 10) as described below:

$$ \begin{gathered} {\text{GroupI }}\left( {{\text{Control}}} \right):{\text{ Normal control mice}}\, + \,{\text{Distilled water}} \hfill \\ {\text{GroupII }}\left( {{\text{Model}}} \right):{\text{ T1DM model mice}}\, + \,{\text{Distilled water}} \hfill \\ {\text{GroupIII }}\left( {{\text{Metformin}}} \right):{\text{ T1DM model mice}}\, + \,{\text{metformin HCl }}\left( {{2}00 {\text{mg}}/{\text{kg body weight}}} \right) \hfill \\ {\text{GroupIV }}\left( {{\text{DDP}} - {\text{Low}}} \right):{\text{ T1DM model mice}}\, + \,{\text{low}} - {\text{dose of CDDP }}\left( {{2}00 {\text{mg}}/{\text{kg body weight}}} \right) \hfill \\ {\text{GroupV }}\left( {{\text{DDP}} - {\text{Med}}} \right):{\text{ T1DM model mice}}\, + \,{\text{medium}} - {\text{dose of CDDP }}\left( {{4}00 {\text{mg}}/{\text{kg body weight}}} \right) \hfill \\ {\text{GroupVI }}\left( {{\text{DDP}} - {\text{High}}} \right):{\text{ T1DM model mice}}\, + \,{\text{high}} - {\text{dose of CDDP }}({6}00 {\text{mg}}/{\text{kg body weight}} \hfill \\ \end{gathered} $$

Throughout the experiment no death and abnormality were observed among all the grouped mice. All the mice (n = 50) induced with multiple low doses of STZ injection showed increased fasting blood glucose levels after 72-h of post STZ doses. Their blood glucose levels were higher than 250 mg/dL. None of the grouped mice showed fed glycemia below 200 mg/dL. Afterwards, the mice in all group were orally gavage with the mentioned doses of crude polysaccharide and metformin, freshly prepared in distilled water once per day according to the body weight of mice for 4-weeks. During the study duration, the food consumption and water intake were recorded daily whereas the body weights, fasting blood glucose levels and feces of all grouped mice were recorded/collected on completion of 1^st^, 2nd, 3rd and 4th week of crude polysaccharide (CDDP) treatment. Later, feces samples were proceeded to extract whole genome for 16S rRNA Illumina Sequencing. At the end of 4-weeks of the treatment, mice were euthanized by cervical dislocation, following by collection of blood samples by excision of eyeball, centrifuged at 4000 rpm for 15 min at 4 ℃ to obtain the serum and freeze at -80 ℃ until further processed. Small portion of colon tissue samples were washed with cold 0.9% saline and then immersed in 4% formalin for histopathological examination.

## Oral Glucose Tolerance Test (OGTT)

Oral Glucose Tolerance Test (OGTT) was performed by following reported protocols [2, 57, 95]. Succinctly, at the end of 4-weeks of oral administration of crude polysaccharide (CDDP), the mice were fasted overnight, and fasting blood glucose levels were recorded. All the mice (n = 6 per group) were orally gavage with 2.0 g/kg body weight of glucose (dissolved in distilled water) and 2-h postprandial blood glucose [PBG-2 h] were recorded using a standard glucometer (Sinocare Inc., Hunan, China). Area under the curve (AUC) were calculated according to the Eq. (1):

1 $${\text{Area under the curve }}=\left( {{\text{basal glycemia }}0~{\text{min}} + {\text{glycemia 3}}0~{\text{min}}} \right) \times 0.{\text{25}}+\left( {{\text{glycemia 3}}0~{\text{min}} + {\text{glycemia 6}}0~{\text{min}}} \right) \times 0.25 +\left( {{\text{glycemia 6}}0~{\text{min}} + {\text{glycemia 12}}0~{\text{min}}} \right) \times 0.5$$

### Measurement of serum insulin level and homeostatic model assessment (HOMA) on HOMA-IR and HOMA-β-cell function

The fasting serum insulin level for the mice in each group (n = 6 per group) were determined by commercially available ELISA (Enzyme-linked immunosorbent assay) kit (Shanghai Lengton Bioscience Co, Ltd., Shanghai, China) as per manufacturer instructions. Furthermore, insulin resistance (HOMA-IR) and β-cells function (HOMA-β-cell function) were calculated according to the studies conducted previously by using the reported formulas (2) and (3) [24, 52, 96–98]:

2 $$ {\text{HOMA}} - {\text{IR }} = \frac{{[({\text{Fasting glucose }}({\text{mmol}}/{\text{L}}) \, \times {\text{ Fasting insulin }}({\text{mU}}/{\text{L}})]}}{2.5} \, $$

3 $$ {\text{HOMA}} - \beta - {\text{cell function }} = \, \frac{{\left[ {20 \, \times {\text{ Fasting insulin }}\left( {{\text{mU}}/{\text{L}}} \right)} \right] }}{{\left[ {\left( {{\text{Fasting glucose }}\left( {{\text{mg}}/{\text{dL}}} \right) \, {-} \, 3.5} \right)} \right]}} $$

### Serum lipid profile analysis

Serum lipid profile including triglyceride (TG), total cholesterol (TC), low-density lipoprotein cholesterol (LDL-C) and high-density lipoprotein cholesterol (HDL-C) for the mice in each treatment groups (n = 6 per group) were determined by using HITACHI 7600 automatic biochemistry analyzer (Hitachi High-Technologies Co., Tokyo, Japan).

### qPCR (quantitative real time PCR) analysis

Proinflammatory cytokines level in intestinal tissue includes interleukin-1β (IL-1β), interleukin-2 (IL-2), interleukin-6 (IL-6), tumour necrosis factor-alpha (TNF-α) and interferon-gamma (IFN-γ) were determined by qPCR. The RNA was extracted by using Total RNA Extraction kit (Vazyme, biotech co. ltd.) as per manufacturer instructions and kept at -80 ℃. The transcription of complementary DNA (cDNA) was done by using commercial kits of HiScript II Q RT Supermix (Vazyme, biotech co. ltd.). The qPCR was executed in Bioer light gene 9600 analyzers using ChamQ SYBR qPCR mastermix kit (Vazyme, biotech co. ltd.). The sequences of Primers were listed in Additional file 1: Table: S2. Each sample were run in triplicates. The expression was calculated and examined with software gene 9600 and differences among the groups were analysed by GraphPad prism 7. Additionally, the proinflammatory cytokines level in serum includes IL-1β, IL-2, IL-6, TNF-α and IFN-γ were quantified by using commercially available Mouse ELISA (Enzyme-linked immunosorbent assay) kits (Shanghai Lengton Bioscience Co, Ltd., Shanghai, China) as per manufacturer instructions. Reading of ELISA were taken on ELISA Plate Reader with all blanks, standards and samples run in duplicates.

### Determination of serum super oxide dismutase (SOD) and malondialdehyde (MDA*)*

The oxidative stress markers: SOD and MDA activity were measured from serum according to manufacturer instructions. These biomolecules were quantified by using commercially available test kits obtained from Solarbio Life Sciences (catalog no. BC0170) and ELISA (Enzyme-linked immunosorbent assay) kit (Shanghai Lengton Bioscience Co, Ltd., Shanghai, China) respectively.

### Histopathological examination

Colon of mice (n = 5 per group) was eviscerated carefully, cleaned, and immersed in 4% paraformaldehyde for 24 h and transferred to the Pathology Department, Dalian Medical University, Dalian, China for processing. Concisely, samples are dehydrated in an ethanol–water gradient, cleared through xylene, and imbed in paraffin. Around, 3 µm thick sections were cut using a rotary microtome (Thermo, Waltham, MA, USA) and stained with hematoxylin and eosin (H&E) dye after deparaffinized in xylene by decreasing ethanol gradients (100, 95, 70 and 50%) and lastly with double distilled water. The stained tissues were then observed under 200X and 400X magnification. Images were captured with Image-PRO Plus software.

### Immunohistochemistr*y*

The expression of Mucin-2 (MUC-2) and Zonula occludens-1 (ZO-1) in colon were analyzed by using immunohistochemistry (IHC). Precisely, the 3 µm sections of colon tissues were cut from paraffin block, deparaffinized using xylene, rehydrated with ethanol gradients and incubated with 3% H_2_O_2_ (Sangon Biotech Co. Ltd. Shanghai, China) for duration of 10 min. The tissue slides were then proceeded for antigen retrieval, the slides were heated in antigen retrieval buffer (Na^+2^ EDTA, pH 8.0) and incubated with primary antibody, rabbit-polyclonal anti-mouse MUC-2 (catalog no. PB0156, 1:100) and ZO-1 (catalog no. 21773–1-AP, 1:200), overnight at 4 ℃. The slides were perfused with horseradish peroxidase-conjugated (HRP) secondary antibody (catalog no. SP-9001) at room temperature for 1 h, washed by phosphate-buffered saline (3 times) and stained with 3,3-iaminobenzidine (DAB) substrate chromogen system (catalog no. ZLI-9018) for 1–5 min; slides were counterstain with hematoxylin, mounted and observed under light microscope at a magnification of 20X, 100 µm scalebar.

### Western blotting

Tissues from colon were used for the extraction of total protein by RIPA (Radioimmunoprecipitation) lysis assay buffer (Proteintech, Wuhan, China) along with protease inhibitor cocktail (Transgene, Biotech, Beijing, China) for 30 min on ice. Later, cell lysate was centrifuged at 12000 g for 5 min at 4 ℃, quantified for protein content by BCA protein assay kit (TransGen Biotech Co, Ltd., Beijing, China) as per manufacturer instructions. A total of 30 µg protein lysates were fractionized by sodium dodecyl sulfate-polyacrylaminde gel electrophoresis (SDS-PAGE) (8–12%) and blotted on polyvinylidene difluoride (PVDF) membranes (0.22 µm) (Immobilon TM-P; Millipore, Massachusetts, USA). Following, 5% non-fat milk was used as a blocking buffer, the membranes were blocked for 1.5 h at room temperature using Tris Buffered Saline (TBS-T: 20 mM Tris–HCl (pH 7.5), 150 mM NaCl, 0.1% Tween 20) to avoid non-specific binding and incubated overnight with primary antibodies [Claudin-1 (catalog no. 13050–1-AP, 1:500) and Occludin (catalog no. 13409-1-AP, 1:2000)] at 4 ℃ using β-actin (catalog no. 20536-1-AP, 1:5000) as a control. The membranes were washed with TBS three times, and then incubated with horseradish peroxidase-conjugated (HRP) secondary antibody (catalog no. SA00001-2, 1:5000) for 1.5 h at room temperature. The protein bands were enhanced by using ECL Chemi-luminescent substrate and images were visualized by automated Imaging System (Imager-Bio-Rad, Bio-Rad Laboratories, Inc., Hercules, CA, USA).

### Immunofluorescence staining

Immunofluorescent staining (IF) was used to examine the levels of insulin receptor substrate-1 (*IRS*-1, catalog no. 17509–1-AP, 1:200) expression in the pancreas. The 5 µm section of pancreatic tissue from the paraffin-embedded block was cut and placed on a positive-charged slide. The slides were deparaffinized in xylene and rehydrated using ethanol gradients; followed by treating the slides for 30 min in citrate buffer at 100 watts in microwave for antigen retrieval and then cool for 1 h. Tissue slide were then blocked for 1 h with 3% BSA solution and incubated overnight at 4 ℃ with IRS-1 antibodies. Slides were washed and sections were incubated with Alexa 488-conjugated secondary antibodies for 1 h. DAPI (4′,6-diamidino-2-phenylindole) was used for staining. Images were taken by confocal scanning microscope.

### Stool sampling, DNA extraction and 16S rRNA gene sequencing

At the end of the research study, the stool (feces) samples were collected and immediately stored at − 80 °C until the DNA extraction. The bacterial DNA was extracted by using stool DNA extraction kit (FORGENE) as per manufacturer instructions. The genomic DNA was quantified spectrophotometrically using NanoDrop ND-1000 (Thermo Fisher Scientific, Waltham, MA, USA) and quality was excised by 1% agarose gel electrophoresis. The composition of bacterial community was observed by V3-V4 region of 16S rRNA gene, amplification of specified region was performed by PCR from microbial DNA. The PCR primers pairs were designed as: Forward primer (515 F, 5*'*-GTGCCAGCMGCCGCGGTAA-3*’*) and Reverse primer (806 R, 5*'*-GGACTACHVGGGTWTCTAAT- 3*’*). Briefly, PCR procedure contain DNA template 2 × taq master mix, 10 µM primers and water. The program of the PCR was set as: Hot start at 98 ℃ for 30 s; 25 cycles of denaturation at 98 ℃ for 15 s, annealing at 58 ℃ for 15 s, extension at 72 ℃ for 15 s and final extension at 72 ℃ for 1 min. The PCR products were purified by Agencourt AMPure XPBeads (Beckman Coulter, Indian apol is, IN) and quantified with Pico Greends DNA Assay Kit (Invitrogen, Carlsbad, CA, USA) as per manufacturer instructions and later send for sequencing on Illumina MiSeq platform (GUHE Info technology Co., Ltd, Hangzhou, China). Library Quant kit Illumina GA revised primer-SYBR Fast Universal kit (KAPA, Wilmington, USA) were used for the preparation of Library and sequenced for 600 cycles on Illumina MiSeq using MiSeq Reagent kit (Illumina, San Diego, USA). Furthermore, QIIME (Quantitative Insights of Microbial Ecology) (QIIME, v1.9.0), tool kit was used to process 16S rRNA sequencing data. Low-quality sequences were removed and high-quality data with 97% similarity were regarded as operational taxonomic unit (OUT) by QIIME software, representing the diversity and abundance of different microbial species in the given samples, described as alpha (α) diversity including Shannon diversity, Simpson and Chao1, were performed by using QIIME and R packages (v3.2.0) for evenness and richness index while, beta (β) diversity was visually represented by weighted-UniFrac distance based on principal coordinate analysis (PCoA) and non-metric multidimensional scaling (NMDS).

### Statistical analysis

All the data results were presented as a Mean ± SEM (Standard error of Mean) per group for the mice in the treatment groups. The statistical analysis was performed using GraphPad Prism 7 software (La Jolla, CA, USA). In order to precise the differences between the CDDP treatment groups (DDP-Low, DDP-Med and DDP-High), metformin HCl and model group were significant, the one-way analysis of variance (ANOVA) was used for the significance followed by Tukey’s multiple comparison test with p < 0.05 were accepted as significant significance.

## Results

### Extraction, monosaccharide composition and molecular weight of crude polysaccharide (CDDP)

The crude polysaccharide was extracted from seaweed, *Dictyopteris divaricata,* by water–ethanol method. The extraction yield of crude polysaccharide (CDDP) was calculated to be 17.78% (w/w). The total sugar content was 96% by using phenol-H_2_SO_4_ method and protein content was 1.15% by bicinchoninic acid method. Monosaccharide composition and molecular weight of crude polysaccharide (CDDP) was determined by HPLC (high performance liquid chromatography) as shown in Table 1.

### Effect of crude polysaccharide (CDDP) on body weight, food consumption, and water intake in Streptozotocin (STZ)-induced T1DM mice

The changes in the body weight, food and water intake were represented in Fig. 2A–C. The body weight of control group tends to be higher in both pre- and post-STZ phases (Additional file 1: Table: S1), than all the other groups. Compared with control group, the STZ had effectively reduced the body weight and increased the food consumption and water intake of mice in model group (Model: ~ 9.05%, 40.15% and 22.60% vs Control: 14.09%, 11.31% and 13.51%, p < 0.001, respectively). Nevertheless, treatment with CDDP doses (DDP-Low, DDP-Med and DDP-High) had successfully attenuated the body weight loss and also improved the water/food disbalances after the oral administration for 4-weeks in contrast to model group. In comparison with CDDP groups, high-dose (DDP-High) showed the significant results in weight gain and restoration of food/water intake balances. Meanwhile, antidiabetic drug, metformin group also exhibits its effect in enhancement of body weight and reduction of food and water consumption during the experiment compared with model group. Our results suggest that increase in body weight and decrease in levels of food/water was dependent on CDDP dose ranging from 200 mg/kg to 600 mg/kg body weight.

### Crude polysaccharide (CDDP) depreciate hyperglycemia in STZ-induced T1DM mice

T1DM-associated hyperglycemia causes impaired protein function and glycation which results in many other disease conditions [52]. In order to evaluate hyperglycemic effect of CDDP, the fasting blood glucose and oral glucose tolerance i.e., 2-h postprandial blood glucose (PBG-2 h) levels were measured after multiple low doses of STZ injection and upon CDDP treatment. As indicated in Fig 3A, B, p < 0.0001, compared with the control group, there was a sharp rise in the blood glucose levels of model group throughout the treatment (Model: + 23.11% v/s Control: 19.28%, p < 0.0001). Oral administration of different doses of CDDP such as DDP-Low, DDP-Med and DDP-High had significantly lowered the elevated fasting blood glucose and PBG-2 h levels when compared with model group. Nevertheless, high-dose (DDP-High) dose of CDDP (DDP-High: − 43.20% vs Model: + 23.11%, p < 0.0001), had shown much promising results in lowering the blood glucose levels thus, demonstrating a beneficial outcome as indicated by the curtailed level of area under the curve (AUCs) as compared to model group (Fig. 3C, p < 0.001). Simultaneously, metformin group had also showed decrease in the blood glucose level when compared with model group (Metformin: -26.87% vs Model: + 23.11%, p = 0.0001).

### Serum Insulin, HOMA-IR and HOMA-β cell function

Deficiency of insulin is often associated with hypertriglyceridemia and hypercholesterolemia [53]. Therefore, the effect of serum insulin level was investigated in all group mice after 4-weeks of CDDP treatment. As delineate in Fig. 4A–C, p < 0.001, compared with the control group, a screwy decrease in the serum insulin, HOMA-β-cell function and increase in HOMA-IR levels were observed in model group as a result of loss of β-cells due to STZ induction (Control: 27.46% v/s Model: 17.19% respectively, p = 0.0003). Notably, oral administration of different CDDP doses (DDP-Low, DDP-Med and DDP-High) to STZ-induced T1DM mice had showed an improvement in the levels of serum insulin, HOMA-IR and partially restored the loss of β-cell by elevating the levels of HOMA-β-cell indicating a significant rise in insulin secretion and indicated a dose-dependent effect of crude polysaccharide (CDDP). Similar outcome was observed in metformin treated group which demonstrated the partial restoration of pancreatic β-cells (Metformin: 11.70% v/s Model: 17.19% respectively, p = 0.0124).

### Attenuation of Dyslipidemia in STZ-Induced T1DM mice by CDDP

Levels of TC, TG, LDL-C and HDL-C were summarized and shown in Fig. 5A–D, compared with control group the levels of TC, TG and LDL-C were considerably higher whereas level of HDL-C was decreased in model group. Contradictory, oral administration of CDDP treatments had successfully decreased the levels of TC, TG and LDL-C and increase HDL-C level in T1DM mice. Moreover, low-dose (DDP-Low) of CDDP showed much difference in TG and TC levels (p = 0.0111 and p = 0.0085, respectively), whereas high-dose (DDP-High) of CDDP exhibit improvement in LDL-C and HDL-C levels which extent statistical intent compared with model group (p = 0.0124 and p = 0.0067, respectively). Positive control, metformin group, showed similar results as that of CDDP groups. Cumulatively, these results insinuate that CDDP showed the possible ameliorative effect on serum lipid profile of T1DM mice which can help in the reduction of diabetic risk factors.

### Analysis of pro-inflammatory cytokines by qPCR (quantitative real time PCR) and ELISA

Levels of pro-inflammatory cytokines IL-1β, IL-2, IL-6, TNF-α and IFN-γ were measured in intestinal tissue and serum of STZ-induced T1DM mice after the oral administration CCDP doses for 4-weeks by qPCR and ELISA techniques. The results revealed that the levels of IL-1β, IL-2, IL-6, TNF-α and IFN-γ were significantly higher in model group in contrast with control group, which proclaimed that T1DM is closely associated with inflammation as illustrated in Figs. 6 and 7A–E. However, these levels of cytokines were reduced after the treatment with CDDP doses, particularly the high-dose of CDDP (DDP-High) had effectively lowered the level of pro-inflammatory cytokines when compared with model group. In addition to this, metformin treatment group also demonstrates the reduction in the cytokines level when compared with model group. Therefore, our study outcome exhibits the inhibitory property of CDDP by reducing circulating levels of pro-inflammatory cytokines in intestinal tissue and serum.

### Effect of CDDP on serum SOD and MDA levels in T1DM mice

Levels of oxidative stress markers: SOD and MDA were measured in serum after 4-weeks of oral administration of CDDP doses. Compared with control group, the SOD enzymatic activity were lower and MDA levels were higher in model group. However, oral administration of different CDDP doses (DDP-Low, DDP-Med and DDP-High) to STZ-induced T1DM mice had showed an improvement in the levels of serum SOD and MDA. In contrary, the high-dose (DDP-High) of CDDP significantly enhanced the SOD and suppressed the MDA levels which extent statistical intent compared with model group (p = 0.0072 and p < 0.0001, respectively). Similar results were observed in metformin treated group. All the results were summarized in Fig. 8A and B.

### Effect of CDDP on histology of colon of STZ-induced T1DM

The histology of colon tissue was evaluated in order to see the effect of STZ on model group and its restoration by CDDP and metformin treatment groups, as shown in Fig. 9. The mice in control group demonstrate a normal healthy colon histology. The structure of epithelium and goblet cells were intact and homogenously arranged. The mucosal space is well-defined. On the contrary, induction of STZ had increased the intestinal permeability in model group which demonstrate severe damage to the histology of colon with decrease in the number of goblet cells, the villi are shorter and irregular with reduced mucosal space, infiltration of inflammatory cells and crippled epithelial cells was seen. These changes in morphology had drastically affect the expression of tight junction proteins (TJs) in the epithelium of colon. However, compared to mice of model group, the CDDP doses group (DDP-Low, DDP-Med and DDP-High) and anti-diabetic metformin group had significantly restored the overall features of colon, reduced the inflammatory cells, improved the number of goblet cells, increase the size of the villi and balanced the mucosal space. As compared to CDDP doses, the high-dose (DDP-High) showed the much promising results in the restoration and improvement in colon histomorphology.

### CDDP modulates Mucin-2 (MUC-2) and Zonula occludens protein-1 (ZO-1) expression in the colon of STZ-induced T1DM Mice

In order to examine the gut barrier integrity after STZ-induction and upon its treatment with CDDP, the expression of Mucin-2 (MUC-2) and Zonula occludens protein-1 (ZO-1) were determined by immunohistochemistry (IHC) technique respectively, after 4-weeks of oral administration of CDDP. Mucin-2 is regarded as the main component of gut epithelium secreted by goblet cells [54]. In our study, a reduction was observed in MUC-2 and Zonula occludens protein-1 (ZO-1) expression in model group. The epithelial cells were considerably damaged, the abundance of goblet cells was reduced leading to the destruction of mucus layer, compared to control group. In addition, the CDDP treatment (DDP-Low, DDP-Med and DDP-High) had dramatically increased the thickness of mucus layer, whereby enhanced the expression of MUC-2 and ZO-1 by improving the structure of epithelial cells and replenished the quantity of goblet cells, especially in high-dose (DDP-High) of CDDP compared to model group. Interestingly, metformin group had also showed the similar results in recovering the epithelial and goblet cells, further improve the mucus layer and integrity of intestinal barrier by inducing the expression of ZO-1 and mucins in STZ-induced T1DM mouse model compared to model group (Fig. 10A and B).

### Immunoblotting

We examined the expression of tight junction proteins (TJs) in the colon. The results demonstrated that the model group showed the lower expressions of Occludin and Claudin-1 compared to control group. However, treatment with CDDP modulated and enhanced the expression of tight junction proteins (Occludin and Claudin-1) in CDDP groups (DDP-Low, DDP-Med and DDP-High) in a dose-dependent manner compared to model group. On the hand, metformin group had also improved the expressions of TJs [(Occludin and Claudin-1) when compared with model group (Fig. 11A and B). Altogether, these findings suggested that oral administration of CDDP may improve the integrity of gut barrier in STZ-induced T1DM model.

### Immunofluorescent staining

To investigate the impact of STZ on insulin producing cells in pancreas, the expression of insulin receptor substrate-1 (IRS-1) were determined by immunofluorescent (IF) technique. The model group showed relatively reduced expression of IRS-1 indicating that STZ had severely disrupted the pancreatic structures and decreased the number of β-cells which aids in insulin secretion when compared to control group. Nonetheless, oral administration of different doses of CDDP (DDP-Low, DDP-Med and DDP-High) had partially restored the β-cells mass in the pancreas in turn improved the architectural structures of pancreas, hence, enhanced the expression of IRS-1 protein as compared to model group. In concordance, the high dose (DDP-High) of CDDP demonstrated the significant improvement in the restoration of β-cells and increased the expression of IRS-1 proteins. On the contrary, the metformin group showed the similar results as that of CDDP treatment groups when compared with model group (Fig. 12A and B). Collectively, these results suggest that efficacy of oral treatment of CDDP in ameliorating the STZ-induced T1DM parameters.

### Crude polysaccharide (CDDP) treatment modulates the overall composition of gut microbiota in diabetic mice

Miseq 16S rRNA gene sequencing was performed to determine the microbial composition of gut. Structural analysis was performed in order to monitor and characterize the alteration and changes occurred in STZ-induced T1DM mice and treatment groups [55]. Alpha-diversity indices were measured in order to compare the diversity and species richness among control, STZ-induced T1DM model, CDDP (DDP-Low, DDP-Med and DDP-High) and anti-diabetic drug, metformin treatment groups. A total of 3010520 raw reads and 28101 OTUs (Operational taxonomic units) ranging from 827 to 1673 were procured in our study, among them 2814977 clean tags (OTUs Sequence) were attained from 24 samples through 16S rRNA Illumina MiSequencing analysis. To demonstrate the shared OTUs among different groups, a Venn diagram was created. As shown in Fig. 13A, there were 963 OTUs shared among six groups. Each leaflet pattern illustrates the number of OTUs in the particular group. The coverage index was found to be 1 in all the six groups correspondingly, highlighting that the sequences were detectable in all the samples.

Based on 97% similarity, five metrics were analysed to conclude α-diversity; Shannon, Simpson, Chao1, Observed Species and Rank Abundance. A box-plot was plotted to support the findings of the data indicating higher α-diversity in control group. The result showed Chao1, was significantly reduced while Shannon and Simpson revealed slight changes in the diversity of microbes in model group compared with control group, indicating diversity of species in the model group which was increase due to intraperitoneal injection of STZ (Additional file 1: Figure: S2). As reported by Yin, Ruiyang et al. [56], STZ doesnot cause any effect on Shannon diversity of gut microbiota community. However, compared with model group, the CDDP treatment groups (DDP-Low, DDP-Med and DDP-High) showed partial restoration of the disbalances. Medium-dose (DDP-Med) of CDDP group showed higher restoration of gut microbiota while anti-diabetic drug, metformin group showed much significant results. The richness and diversity in all the six groups were also observed by rank abundance and rarefaction curves of observed species, which showed low species richness and evenness in model (T1DM) group which was modulated by the different doses of CDDP (DDP-Low, DDP-Med and DDP-High) and metformin. Altogether all these results collectively conclude that induction of STZ injection for 5-days at a dose of 50 mg/kg body weight to induce T1DM had effectively reduced the overall microbial population of gut microbiota. Additionally, treatment with CDDP doses (specifically DDP-Med) and metformin helps in partial restoration of overall community of the bacterial population in gut (Fig. 13B, C).

### Effect of β-diversity on microbial diversity in STZ-induced T1DM (model) mice and crude polysaccharide (CDDP) treatment groups

Differences in β-diversity were analysed by PCoA (Principal Coordinates Analysis) and NMDS (Non-metric multidimensional scaling plot) which indicate changes in the community of different samples. Based on the weighted-unifrac distance of PCoAs and NMDS of 16S rRNA sequencing, resulted changes to the population of microbes following the STZ induction, crude polysaccharide (CDDP) and anti-diabetic drug, metformin treatment with control group. Data showed a notable separation of points of model group (blue) which were distant because of induction of T1DM by STZ indicating structural variability, from control group (red), which later cluster together close to control group after the treatment of crude polysaccharide [CDDP] DDP-Low (purple), DDP-Med (orange), DDP-High (yellow) and metformin (green) in PCoA and NMDS. The data was in correspond to the α-diversity and taxonomic data presented in the study (Fig. 14A, B).

### Taxonomical analysis of gut microbiota after STZ-induction and crude polysaccharide (CDDP) treatment

Analysis of taxonomy was based on the assigned sequences which reveal differences statistically significant between groups at the taxonomic levels i.e., phylum, family and genus. At phylum level, the dominant bacteria were *Firmicutes, Bacteroidetes, Proteobacteria* and *Actinobacteria*, detected in all groups. Compared with control group, there was a decrease in the proportion of *Firmicutes* (Model: 33.18% vs. Control: 57.26%) and increase in the proportion of *Bacteroidetes* (Model: 55.92% vs. Control: 36.65%), *Proteobacteria* (Model: 4.64% vs. Control: 3.93%) and *Actinobacteria* (Model: 4.49% vs. Control: 0.66%) were observed in model group. After 4-weeks of oral administration of crude polysaccharide (CDDP) and metformin, compared with model group the ratio of *Firmicutes/Bacteroides, Proteobacteria* and *Actinobacteria* were returned to normal as close to control group (Fig. 15A and Additional file 1: Table: S3).

Moreover, the differences in the microbial population were evaluated among all the treatment groups at family level. The discrepancy in bacterial families was presented in Fig. 15B and Additional file 1: Table: S4. Proportion of S24-7, *Paraprevotellaceae. Odoribacteraceae* and *Corynebacteriaceae* were increased in model group (Model: 35.25%, 8.24%, 6.18% and 5.77% vs. Control: 22.61%, 2.20%, 3.28% and 0.12% respectively), while levels of *Lactobacillaceae, Ruminococaceae, Lachnospiraceae* and *Rikenellaceae* were significantly decreased in model group as compared to control group (Model: 13.60%, 9.00%, 6.35% and 6.84% vs. Control: 38.91%, 9.27%, 8.00% and 7.93% respectively). Interesting the CDDP and metformin treated mice showed significant outcome in the restoration of all the above-mentioned genera when compared with model group indicating that the treatment of crude polysaccharide (CDDP) had a significant role in gut dysbiosis restoration at both phylum and family levels.

At genus level, proportion of *Bacteroides, Corynebacterium, Ruminococcus* and *Parabacteroides* were increased in model group as compared with control group (Model: 35.25%, 8.24%, 6.18% and 5.77% vs. Control: 22.61%, 2.20%, 3.28% and 0.12% respectively). However, there was decrease in the proportion of *Lactobacillus, Prevotella* and *Oscillospira* in model group as compared with control group (Model: 13.60%, 9.00% and 6.35% vs. Control: 38.91%, 9.27% and 8.00% respectively). All these bacteria were restored in CDDP (DDP-Low, DDP-Med and DDP-High) and anti-diabetic drug, metformin treatment groups, especially *Lactobacillus, Bacteroides* and *Parabacteroides*. Heatmap was generated to highlight the dysbiosis (The results are represented in Additional file 1: Figure: S3 and Additional file 1: Table: S5). These outcomes implied that CDDP treatment improved the overall community of intestinal flora in T1DM mice induced with STZ.

## Discussion

Despite the presence of variety of medicinal drugs that can treat diabetes, natural herbs such as plants, that have shown significant therapeutic remedy on the treatment and can be consumed for long-term because of its beneficial outcome, availability, low cost of preparation, minimum or no side effects and less toxicity [3, 28, 57, 58]. On the basis of their functional ability several natural plants such as seaweeds, contain variety of nutrients that are suitable for consumption and its polysaccharides isolated from different plant sources had showed a promising outcome than the standard available drugs to treat diabetes complications such as lowering fasting glucose levels by enhancing insulin secretion, promotes and escalates the growth of intestinal gut microbiota, increases volume of stool and reduces the overall risk of colon cancer in STZ-induced T1DM animals [42, 50, 59–62].

Consistent with the previous literatures, treatment with extract of medicinal plants can revert the effect of β-cell damage caused by STZ via gradually activating β-cells which returned to normal cells after effective treatment. One such plant *Cassia auriculate* L, leaves were reported to lower blood glucose and serum lipid levels in T2DM [58]. Additionally, extract from *Picralima nitida* had shown an anti-diabetic and anti-lipogenic property in vitro as well in vivo system of alloxan-induced diabetic mice [63]. Moreover, a study showed that *Angelica sinensis* polysaccharide was used to treat mice with T2DM by low dose of STZ injection [9]. *O. japonicas* crude polysaccharide showed antagonistic effect in alloxan-induced diabetic mice while its purified form of polysaccharide showed anti-diabetic activity in streptozotocin-induced diabetic rats [64]. Several lines of evidences delineated the experimental affirmation of seaweed polysaccharides in the treatment of different diseases such as obesity, diabetes, allergies, inflammation and hepatic steatosis [65–70]. In concordance with the beneficial effects of polysaccharides our research group isolated the polysaccharide from seaweed, *Dictyopteris divaricata.* In general, the impact of this particular polysaccharides on diabetes related disbalances and gut microbial dysbiosis has been seldomly investigated and studied. In this research our data divulged that the oral supplement of crude polysaccharide *Dictyopteris divaricata* (CDDP) at a dose of 200, 400 and 600 mg/kg body weight (DDP-Low, DDP-Med and DDP-High respectively) for 4-weeks had reverted different parameters of diabetes such as hyperglycemia, hypoinsulinemia, destructive islets morphology, hyperlipidemia, polyphagia, polydipsia, weight loss, downscale intestinal inflammation, rejuvenated the gut microbiota, enhanced the abundance of microbial diversity, ameliorated the mucosal and intestinal barrier integrity and upregulate the expression of insulin producing proteins (IRS-1) in STZ-induced type-1 diabetes mellitus (T1DM) mice along with antidiabetic drug, metformin given orally at a dose of 200 mg/kg body weight which serves as a positive control in our research study.

In the present study, the peak observed by HPLC showed the composition of monosaccharide present in CDDP consisting of nine bioactive compounds such as Mannose, Ribose, Rhamnose, Glucuronic Acid, Glucose, Galactose, Xylose, Arabinose and Fucose, and their percentage content was 15.02%, 9.90%, 1.28%, 17.54%, 1.86%, 17.19%, 4.54%, 0.505% and 32.13% respectively (Additional file 1: Figure: S1). Fucose was the main monosaccharide form found to be 32.13% of the total monosaccharide content in CDDP, as reported in previous study [40]. The molecular weight was found to be 63,060 g/mol (Table 1).

In our study, an insulin-dependent (IDDM) or type-1 diabetes mellitus (T1DM) model induced by STZ was found to be competent of enhancing the blood glucose levels, food and water intake, and reduction in body weight. Altogether the above parameters were the depictions for diabetes development in mice after 72 h of the STZ doses. These inductions lead to the pancreatic islet destruction and apoptosis of β-cells which takes up STZ by glucose transporter, GLUT2 through DNA alkylation [20, 71]. It was clearly evident from our data that the above-mentioned parameters were reverted by oral administration of CDDP in a dose-dependent manner, as a consequence of diet-induced supplement (Figs. 2 and 3).

Additionally, another important effect of CDDP administration was the dramatic increase in the insulin levels, β-cells mass, upraised levels of HDL-C, SOD and reduced levels of TG, TC, LDL-C, pro-inflammatory cytokines and MDA. The overall finding suggests that CDDP may have hypercholesteraemic and hypertriglyceridemic properties. It attributes that the CDDP can be used to revert the hyperlipidiemic conditions in T1DM mice which enhance insulin uptake, resulting in increase of glucose uptake and reducing free fatty acids movements which may meliorate the renovation of pancreatic β-cells [72]. Besides these the increased in the thickness of gut mucus layer was observed in CDDP-treated mice. Altogether these beneficial outcomes were found to be interlinked with the alteration of gut microbiome by CDDP. To our best knowledge, this study is the first direct confirmation showing that the anti-diabetic property of crude polysaccharide (CDDP) is associated with the modulation of intestinal gut microbiota composition (Figs. 4, 5, 6, 7, 8).

In order to investigate the correlation of STZ-induced gut dysbiosis and its restoration upon CDDP treatment on the physiology of host, we explicate the changes occur in histology of colon, inflammatory responses, gut permeability, tight-junction proteins and insulin receptor substrate-1 (IRS-1) expression after the induction of STZ and CDDP treatment doses (DDP-Low, DDP-Med and DDP-High). In considerations with our study, STZ caused intense effect on morphology of pancreas and colon. Particularly, in pancreas there was a dramatic decrease in the β-cells number which contribute towards the lower IRS-1 expression in the pancreas ultimately leading to low levels of insulin secretion (Fig. 12). Concurrently, oral doses of CDDP had improved and restored the overall pancreas histology with an increased in the number of β-cells required for insulin production and restored the morphology. On the other hand, STZ had disturbed the permeability of intestine. The histological examination of colon revealed the loss of goblet cells, disrupted mucosal space, markedly reduced villi length, infiltration of inflammatory cells and loss of epithelial cells in STZ-induced T1DM model group while CDDP (DDP-Low, DDP-Med and DDP-High) supplement improved the gut morphology and minimizes its adverse effect after treatment (Fig. 9). These goblet cells play a vital role in maintaining the gut intestinal barrier, its permeability, keeping the intestinal structures intact and provide protection from harmful chemicals or pathogens [73].

To further validate the modulation of dietary supplements including polysaccharides used to enhance gut integrity, we examined Mucin-2 (MUC-2) and tight junction proteins (TJs) i.e., cytoplasmic scaffolding protein (Zonula occludens-1, ZO-1) and transmembrane barrier proteins (i.e., Claudins-1 and Occludin) expressions by immunohistochemistry (IHC) and western blot technique, which plays an integral role in balancing gut barrier permeability and protection against detrimental microbes which disturbed the gut homeostasis [74–77].. All these proteins not only maintain the gut integrity but also regulates tight junction complex by keeping the cells connected and intact alongside ZO-1 [78–80]. It was found that STZ-induction had caused effect on mucin producing goblet cells which disrupted the mucosal layer whereby, decreasing the MUC-2 expressions and curtailed the expression of tight junction proteins in intestine of model group, particularly in Claudin-1 which evidently showed lower Occludin expression, as these are corelated with higher levels of proinflammatory cytokines (including, IL-1β, IFN-γ and TNF-α) [81]. In general, this could only be possible because of continuous localization and remodelling of tight junction proteins [82]. Contrastingly, CDDP (DDP-Low, DDP-Med and DDP-High) administration restored the mucin producing goblet cells and tight junction protein structures which eventually enhanced MUC-2, Claudins-1 and Occludin and ZO-1 expressions by improving intestinal barrier integrity and meliorate colon structure (Figs. 10, 11).

In order to further evaluate the supplementary property of CDDP, we investigated its effect on gut microbial disturbances. Thereby, shared and treatment-groups OTUs were illustrated in Venn Diagram (Fig. 13A). Additionally, the current study showed the first time dysbiosis of bacterial diversity in the gut after STZ-induction and treatment with CDDP by high-throughput next-generation 16S rRNA Illumina MiSeq sequencing. Cumulative evidences support that majority of prebiotics aids in providing the beneficial effect on gut. Thus, contributing to become an important part of the biological barrier of gut as these gut microbiota plays an important role in our body system [32]. As mentioned previously, one known polysaccharide of marine algae, *Sargassum confusum* has been studied and used to cure many known diseases. The genus also showed improvement in balancing gut flora which was affected by T2DM and obesity [83]. For this purpose, α-diversity, β-diversity were studied to investigate the notable differences in the distribution and richness of microbial gut flora among treatment groups. Hence, significantly separated clusters of gut microbiome and α-diversity exhibit that STZ-induced model group showed the lower species richness and evenness that was enhanced and augmented after oral treatment of CDDP for 4-weeks. Our study showed no difference in alpha diversity indices such as Shannon and Simpson as the data showed no changes in the composition of gut microbiota (Figs. 13, 14 and Additional file 1: Figure: S2). These findings imply that crude polysaccharide-treated mice had showed higher species abundance and richness, suggesting that oral administration exhibited positive harmonizing effect on gut microbial flora that was disturbed because of STZ-induction.

In order to investigate, the abundance of bacterial population at different levels of taxonomy, taxonomic study is prosecuted by 16S rRNA sequencing to distinguish the composition of different flora in our study at phylum, family and genus level. The results of 16S rRNA gene sequencing at phylum level showed that STZ-induced T1DM had adversely affected the abundance of microorganisms in gut. The proportion of *Firmicutes* and *Bacteroidetes* were balanced in control group. Meanwhile, in T1DM model group, we found an increase in *Bacteroidetes* and decrease in *Firmicutes* abundance, correspondingly. Accumulating literature had shown that the ratio of these two phyla; *Firmicutes* and *Bacteroidetes* were increased in people and animals having diabetes [84–86]. In mice it was stated that T1DM was altered with diversity and composition of gut microbes, reduction in the ratio of *Firmicutes* and *Bacteroidetes*, increased *Proteobacteria* and reduced butyrate-producing bacteria subgroups. This dysbiosis coincides with intestinal permeability, translocation of microbial materials through epithelium and increased aberrant presentation of foreign and self-antigens, activating proinflammatory cytokines in the gut and pancreas [31, 33, 34]. Interestingly, our study reports that CDDP (DDP-Low, DDP-Med and DDP-High) treatment plays a rudimentary role in reverting the gut dysbiosis at phylum level as similar like control group (Fig. 15A and Additional file 1: Table: S3). An increased level of S24-7, *Bacteroidaceae* and *Paraprevotellaceae* and decreased level of *Lactobacillaceae, Rumminococaceae, Lachnospiraceae* and *Rickenellaceae* was observed in T1DM-model group at family level. Previous studies reported that high levels of TC, TG, HOMA-IR, blood glucose, inflammation and LPS was often because of increase proportion of S24-7, *Bacteroidaeeae* and *Prevotella* genera [84, 87] and decrease in genus *Lactobacillaceae, Ruminococcus* and *Lachnospiraceae* at family level in model group compared with control group, were associated with animals and patients suffering from diabetes, specifically T1DM [17, 83, 88]. Consistent with the previous literatures, our study reported the similar outcomes. The notable improvement was seen after treatment with CDDP doses (DDP-Low, DDP-Med and DDP-High), presented that the reduction of some bacteria at family level, are associated with lowering of glucose levels such as S24-7, *Prevotella* and *Bacteroidaceae* and increase of *Lactobacillus sp.* and *Ruminococcaceae* (Fig. 15B and Additional file 1: Table: S4). Nonetheless, at genus level, the pathogenic flora such as *Bacteroidetes, Corynebacterium* and *Parabacteroides* were enhanced in model group. On contrary, the health beneficial flora such as *Lactobacillus* was enriched after CDDP treatment (DDP-Low, DDP-Med and DDP-High) (Additional file 1: Figure/Table: S3/S5). Collectively, all these outcome verdicts that diet is considered as the most important factor in changing the gut flora. Therefore, dietary supplement of seaweed polysaccharide (CDDP) not only helps in ameliorating the abundance of gut flora but also aids in the reinstatement of beneficial gut microbiota such as *Ruminococcaceae* (butyrate-producing bacteria) and *Lactobacillaceae* (Lactic-acid producing bacteria) similarly decreasing the non-beneficial bacteria such as Bacteroidetes, Parabacteroides and Proteobacteria, which suggest that CDDP is safe to be given orally in patients with perturbed gut microbiota.

## Conclusions

For the first time we demonstrate the effect of crude polysaccharide (CDDP) extract from the seaweed, *Dictyopteris divaricata.* Our study outcome suggested its beneficial effects not only on hyperglycemia, body weight, insulin and dyslipidemia but also aid in attenuating gut microbiota and maintaining gut barrier integrity. However, its deeper mechanisms are still remained unstudied and unexplored. Our study substantiates antihyperglycemic, anti-inflammatory and antilipidemic property of CDDP in T1DM mice. However, the promising effects were observed in high-dose of CDDP when given orally for 4-weeks, which ameliorate the symptoms of T1DM in STZ-induced mice. In consideration of our research, all these factors demonstrates that the outcome of this crude polysaccharide (CDDP) is found to be effective and non-toxic therefore, it can be used as a functional food for T1DM patients as a source of naturally found antidiabetic drug and can be used to develop drug for diabetic treatment to overcome the metabolic disorders. Although, more research needs to be done before human usage in order to know its pharmaceutical approaches, bioactivities of crude polysaccharide and study of its mechanical pathway that leads to the reduction of blood glucose by oral dosing of crude polysaccharide (CDDP).

**Table 1:** Monosaccharide composition and molecular weight of crude polysaccharide (CDDP) from seaweed, *Dictyopteris divaricata* by HPLC

Component	Mg/kg	%
Monosaccharide composition		
Mannose	4397.62	15.02
Ribose	2900.62	9.90
Rhamnose	373.64	1.28
Glucuronic Acid	5135.51	17.54
Galacturonic Acid	ND	ND
Glucose	545.87	1.86
Galactose	5035.28	17.19
Xylose	1328.32	4.54
Arabinose	160.42	0.55
Fucose	9408.04	32.13
Molecular weight		
Mw (g/mol)	6.306 × 10^4^	63,060
Mn (g/mol)	5.647 × 10^4^	56,470
Mp (g/mol)	4.918 × 10^4^	49,180
Mz (g/mol)	3.462 × 10^5^	346,200
Mw/Mn	1.117	1.117
Mz/Mn	6.131	6.131

## Supplementary Information

**Additional file 1: Figure S1**. High performance liquid chromatography (HPLC) analysis of crude polysaccharide (CDDP) from seaweed,* Dictyopteris divaricata*.; **Figure S2**. Box-plot of Shannon index (0.0231), Simpson index (0.0154) and Chao1 (0.0456) was plotted to observe the changes in species richness and evenness; **Figure S3**. Heatmap of different taxa at genus level. Data represents the degree of similarity among the groups in the form of cluster and dissimilarity arranged individually. Data is a representative of twenty-four samples; **Table S1**. The body weight of the mice in the experimental study. **Table S2**. List of qPCR primers; **Table S3**. The relative abundance (%) of all the phyla at phylum level; **Table S4**. The differences (%) between the proportion of bacteria at family level; **Table S5**. Representation of bacterial microbial community (%) at genus level.

## Data Availability

The original data for this work are available upon email request to the corresponding author.
