# Supplementary material for: Effect of crude polysaccharide from seaweed, Dictyopteris divaricata (CDDP) on gut microbiota restoration and anti-diabetic activity in streptozotocin (STZ)-induced T1DM mice
Source: Gut Pathog. 2022 Sep 17;14:39. doi: 10.1186/s13099-022-00512-1 (PMC9482207; doi:10.1186/s13099-022-00512-1)
Supplement: Supplementary file 1 — Additional file 1: Figure S1. High performance liquid chromatography (HPLC) analysis of crude polysaccharide (CDDP) from seaweed, Dictyopteris divaricata.; Figure S2. Box-plot of Shannon index (0.0231), Simpson index (0.0154) and Chao1 (0.0456) was plotted to observe the changes in species richness and evenness; Figure S3. Heatmap of different taxa at genus level. Data represents the degree of similarity among the groups in the form of cluster and dissimilarity arranged individually. Data is a representative of twenty-four samples; Table S1. The body weight of the mice in the experimental study. Table S2. List of qPCR primers; Table S3. The relative abundance (%) of all the phyla at phylum level; Table S4. The differences (%) between the proportion of bacteria at family level; Table S5. Representation of bacterial microbial community (%) at genus level. [file 13099_2022_512_MOESM1_ESM.docx]

**SUPPLEMENTARY FIGURES & TABLES**

**Supplementary Table: S1**. The body weight of the mice in the experimental study.

| **Group** | **Initial Weight**  **(g)** | **Weight after STZ injection**  **(g)** | **Changes in body weight (%)** |  |  |  |
| --- | --- | --- | --- | --- | --- | --- |
|  | **Mean±SEM** | **Mean±SEM** |  |  |  |  |
| **Control**  **(n=10)** | 19.51 ± 0.27 | 21.39 ± 0.34 | 10.72 | **Body Weight Gain** | | |
| **Model (T1DM)**  **(n=50)** | 20.56 ± 0.14 | 19.63 ± 0.16 | -5.30 | **Body Weight Loss** | | |

**
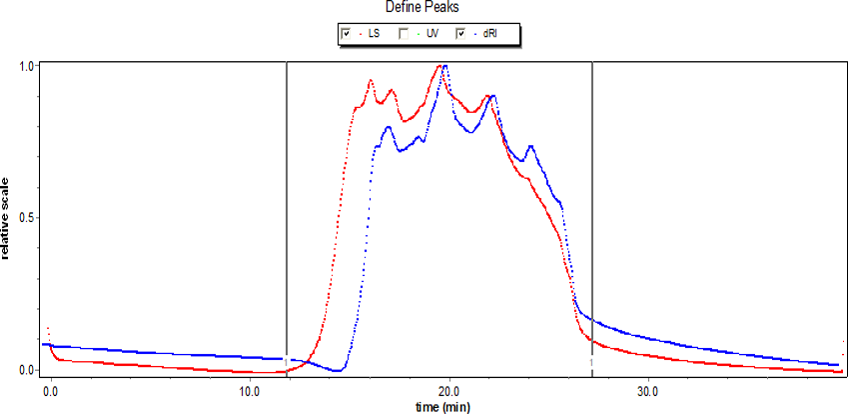
**

**Supplementary Figure: S1.** High performance liquid chromatography (HPLC) analysis of crude polysaccharide (CDDP) from seaweed, *Dictyopteris divaricata.*


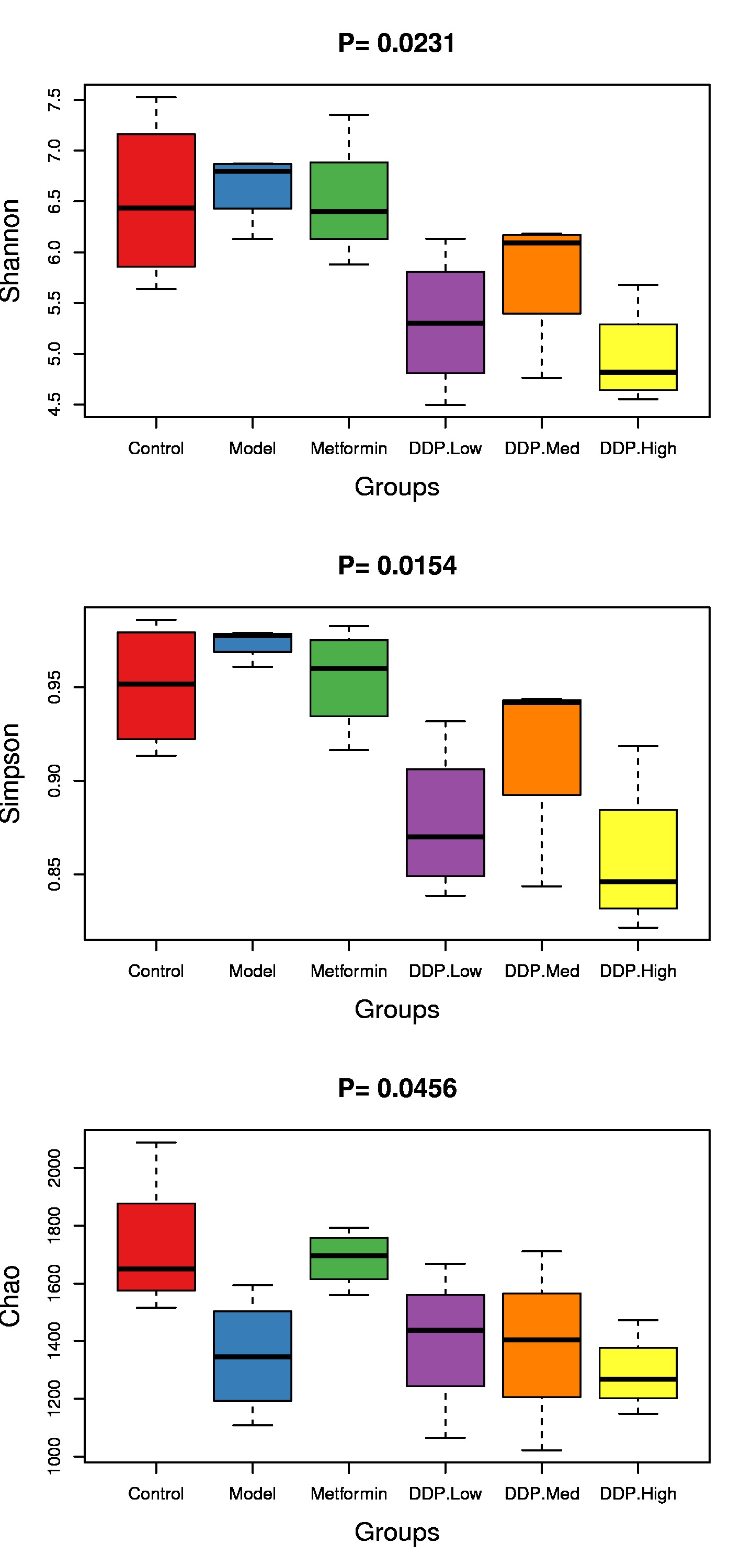


**Supplementary Figure: S2.** Box-plot of Shannon index (0.0231), Simpson index (0.0154) and Chao1 (0.0456) was plotted to observe the changes in species richness and evenness.

**Supplementary Table: S2.** List of Primers used for qPCR.

| Cytokines | Primer | Sequence (5' - 3') |
| --- | --- | --- |
| IL-1β | F | GAAATGCCACCTTTTGACAGTG |
|  | R | CTGGATGCTCTCATCAGGACA |
| IL-2 | F | CCTGAGCAGGATGGAGAATTACA |
|  | R | TCCAGAACATGCCGCAGAG |
| IL-6 | F | GAGGATACCACTCCCAACAGACC |
|  | R | AAGTGCATCATCGTTGTTCATACA |
| TNF-α | F | CATCTTCTCAAAATTCGAGTGACAA |
|  | R | TGGGAGTAGACAAGGTACAACCC |
| IFN-γ | F | TCAAGTGGCATAGATGTGGAAGAA |
|  | R | TGGCTGTGCAGGATTTTCATG |

***^*^*^F = Forward Primer^ *^*^*^R = Reverse Primer^**

**Supplementary Table: S3.** The relative abundance (%) of all the phyla at phylum level.


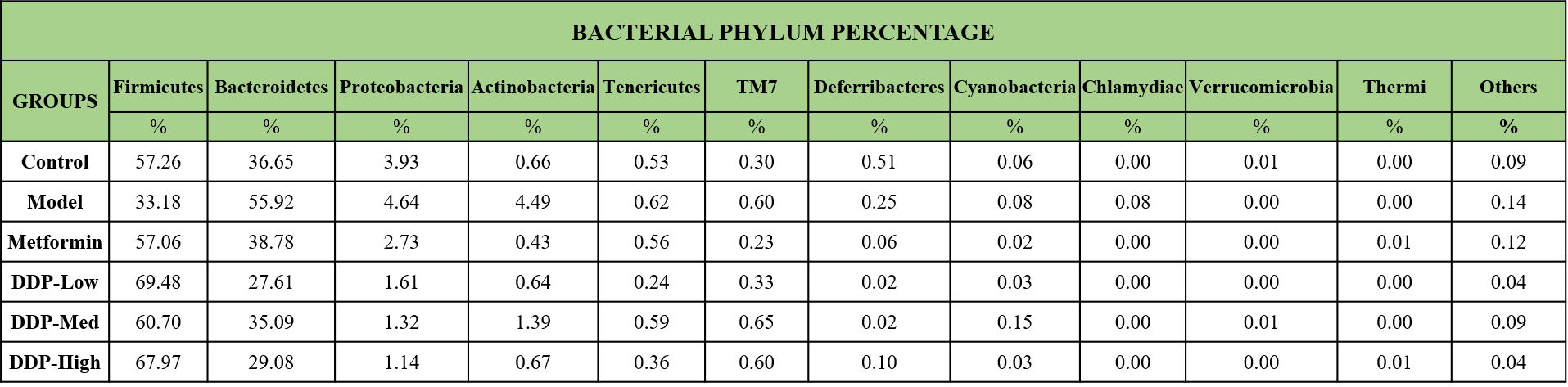


**Supplementary Table: S4.** The differences (%) between the proportion of bacteria at family level.


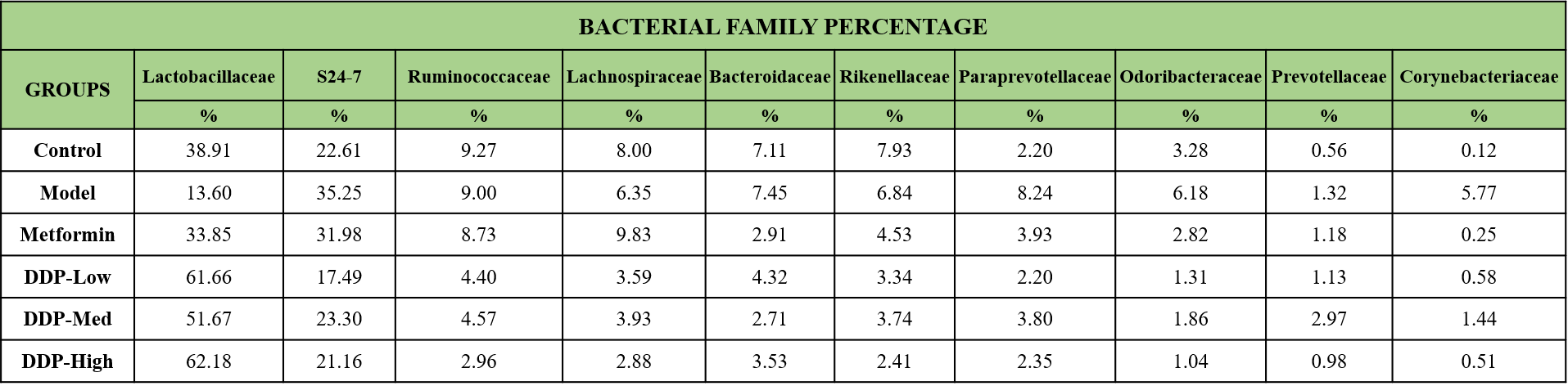


**Supplementary table: S5.** Representation of bacterial microbial community (%) at genus level.

**
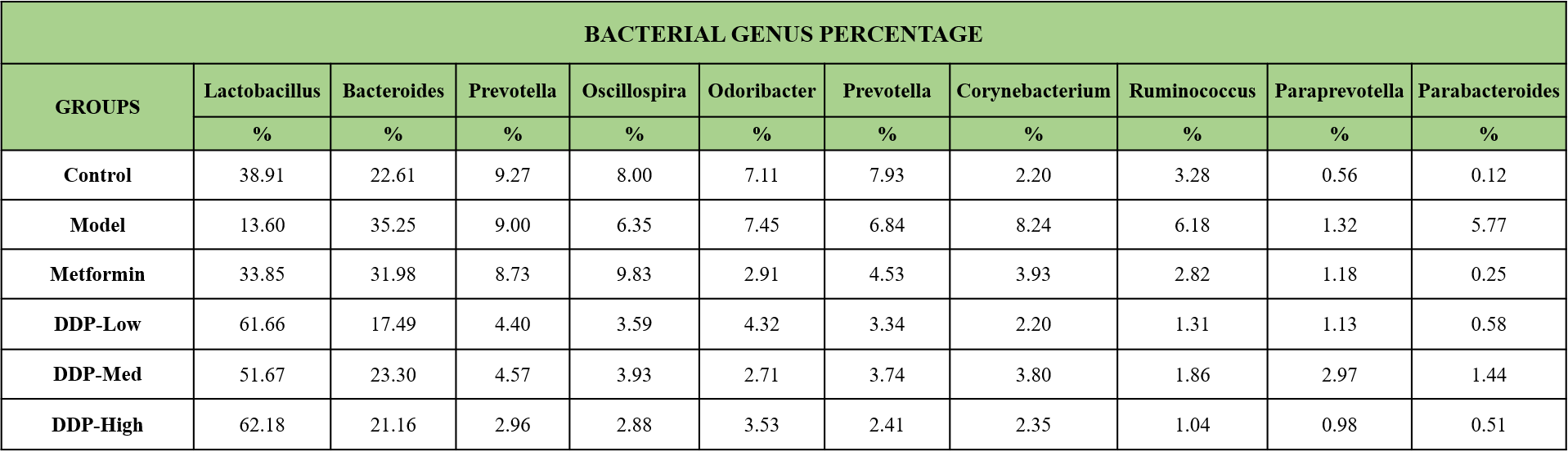
**

**
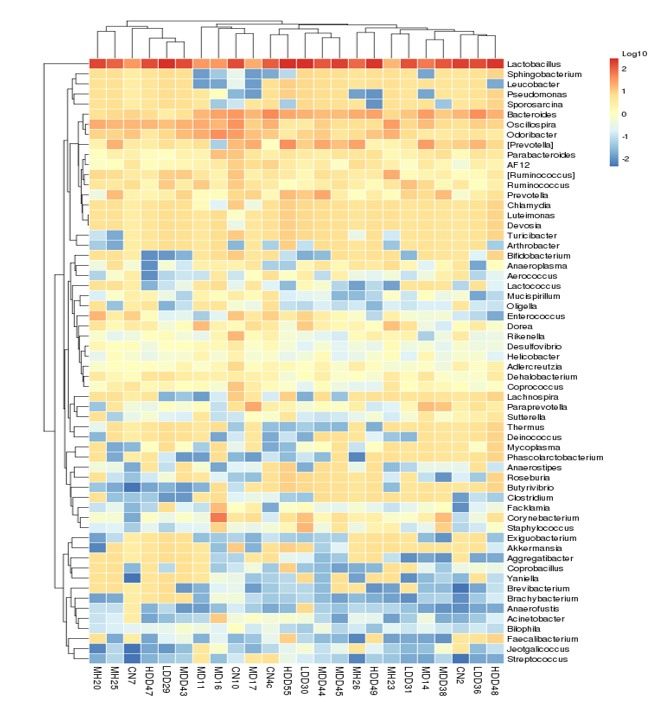
**

**Supplementary Figure: S3. Heatmap of different taxa at genus level.** Data represents the degree of similarity among the groups in the form of cluster and dissimilarity arranged individually. Data is a representative of twenty-four samples.
